# Beyond a solvent: triple roles of dimethylformamide in organic chemistry

**DOI:** 10.1039/c8ra04985h

**Published:** 2018-08-03

**Authors:** Majid M. Heravi, Mahdieh Ghavidel, Leyla Mohammadkhani

**Affiliations:** Department of Chemistry, School of Sciences, Alzahra University Vanak Tehran Iran mmh1331@yahoo.com mmheravi@alzahra.ac.ir

## Abstract

*N*,*N*-Dimethylformamide (DMF) is frequently used as an aprotic solvent in chemical transformations in laboratories of academia as well as in those of chemical industry. In the present review, we will reveal that DMF is actually something much more than a solvent. It is a unique chemical since, as well as being an effective polar aprotic solvent, it can play three other important roles in organic chemistry. It can be used as a reagent, a catalyst, and a stabilizer.

## Abstract 2

*N*,*N*-Dimethylformamide (DMF) is frequently used as an aprotic solvent in chemical transformations. It is a multipurpose compound besides being an effective polar aprotic solvent. DMF can be act as a reagent, a catalyst and a stabilizer.

## Graphical abstract

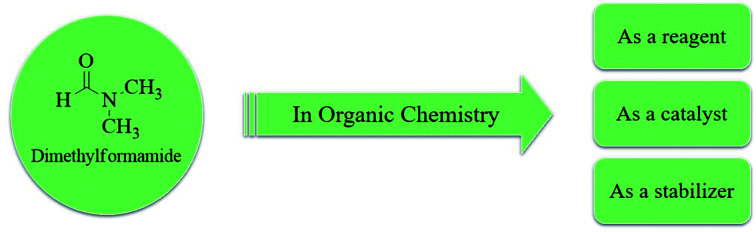

## 1. Introduction

*N*,*N*-Dimethylformamide (DMF) is an extraordinary organic compound with the formula (CH_3_)_2_NC(O)H. It generally abbreviated and literally always called, DMF (should not be mistaken with dimethylfuran or dimethyl fumarate). It is a colourless liquid with a high boiling point which is miscible with water and also with a majority of common organic solvents. DMF should be naturally odorless, however in its technical grade or degraded samples every so often it has a fishy smell due to some impurity of dimethylamine.^1,2^

As its name implies, it is a formamide derivative. Although, among most chemists, DMF is known as an inexpensive commercially available solvent which is widely used in synthetic organic chemistry, by a second look and further consideration, it is actually more than a solvent, showing multipurpose applications. It is a polar aprotic, but hydrophilic, solvent that dissolves most organic compounds. Thus, it is especially useful in organic reactions when their mechanisms involve polar species, such as SN_2_ reactions.^3^ Therefore, DMF is mostly used as an excellent polar solvent with a relatively low evaporation rate, useful for a wide variety of organic transformations. Its usefulness and superiority as a solvent is due to its favored dissolution actually provided by favorited interactions with a substrate.

This uniquely versatile and powerful chemical is frequently used as a solvent for preparation of colloids,^4–6^ synthesis of block-copolymers,^7,8^ and also many other types of organic reactions^9^ like solvent-free hydrolysis, allylation, decarboxylation, polymerization, *etc.*^10–15^ It is also found as a perfect solvent for peptide coupling in the pharmaceuticals industry, in development and production of pesticides and in the manufacture of adhesives, synthetic leathers, fibers, films, and surface coatings. It also is used in the manufacturing of dyes as an important raw material which is consumed during a reaction.^16^ DMF also is a common solvent utilized in electrospinning.^17,18^

Beyond being used as an useful solvent, DMF is employed as a reagent in some important organic reactions, such as the Vilsmeier–Haack reaction,^19^ which results in the formylation of aromatic, non-aromatic, and heteroaromatic compounds.^19–30^ DMF can also be used as a reagent in two other important organic name reactions such as the Friedel–Crafts reaction^31^ and Beckmann rearrangement.^32,33^

**Fig. 1:**
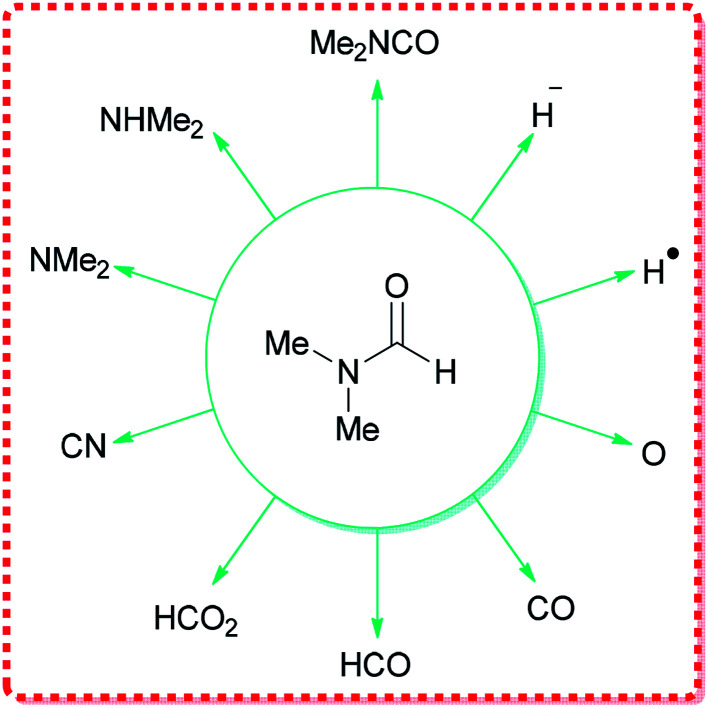
Various functional groups that can be derived from DMF.

DMF can react as either an electrophilic or a nucleophilic agent, thus, can be considered as the source of various key intermediates mediating a plethora of important reactions.^34^ More significantly, because of its structure, DMF can participate in many reactions by serving as a multipurpose building block for various units, such as HCO_2_, O, CO, H˙, H^−^, NMe_2_, CONMe_2_, Me, CHO, *etc.* (Fig. 1).

It is also a common catalyst^35–38^ used in the synthesis of acyl halides, in particular, and in the synthesis of acyl chloride from carboxylic acids using oxalyl or thionyl chloride. The catalytic mechanism entails reversible formation of an imidoyl chloride.^39,40^ It acts as a catalyst in preparing the corresponding azepine,^12^ for the cycloaddition reaction of CO_2_ to propylene oxide,^41,42^ synthesis of cyclic carbonates,^43^ condensation reactions of alkylisocyanides (or arylisocyanides) with barbituric acid derivatives,^13^ reductive ring-cleavage of isoxazole,^10^ allylation of aldehydes,^44^ conversion of both primary and secondary alcohols to bromides,^45^ and acylation of aromatic compounds.^46^

It was disclosed that DMF also can act as a stabilizer in some reactions. In this regard, synthesis of DMF-stabilized metal nanoclusters and nanoparticles were reported.^47–51^ Copper (Cu),^52^ iridium (Ir),^53^ gold (Au),^54^ palladium (Pd),^55^ silver (Ag),^56^ and Fe_2_O_3_ (ref. 57) nanoparticles are stabilized with DMF molecules through interaction of amido groups of DMF with NCs.

In addition, DMF usually serves as a solvent in the formation of peptide-bonds.^58^ DMF dissolves amino acids and coupling reagents and does not react with piperidine, but this reagent can jeopardize peptide synthesis by decomposition into formaldehyde (HCHO) and dimethylamine (HNMe_2_).^59^

Worthy of mention is that on the basis of published reports by NIOSH (The National Institute for Occupational Safety and Health), DMF is readily absorbed through the skin or by inhalation or ingestion. This chemical is a potent liver toxin. DMF is also known to cause constipation, abdominal pain, vomiting and nausea, headache, weakness, dizziness, skin problems, and alcohol intolerance.^60^

The solvent effect of DMF on the efficiency and solvation of anions was reviewed by Parker in 1962.^61^ Other significant functions of DMF, such as being utilized as a ligand, reducing agent, and dehydrating agent were reported by Muzart and co-workers in 2009.^34^ In addition, recent developments in the applications of DMF in the area of nitrilation, amination, formylation, aminocarbonylation, and amidation, as well as its reaction with arynes was reviewed in 2012;^62^ thus, these are not deliberated herein. As a matter of fact, the goal of this report is to disclose other roles of this unique chemical as reagent, catalyst, and stabilizer which have not been comprehensively reviewed. Herein, we try to provide an accessible reference and attract the attention of chemists and stir up their interest in using DMF in organic synthesis and trying to open other new gateway for more applications of this extraordinary compound as a unique and multipurpose chemical. We also frequently used DMF as an efficient solvent in our laboratory.^63–65^

Being thoughtful about this remarkable chemical, in this report we focus on the applicability of DMF as reagent, catalyst, and stabilizer.

## 2. DMF as a reagent

**Scheme 1:**
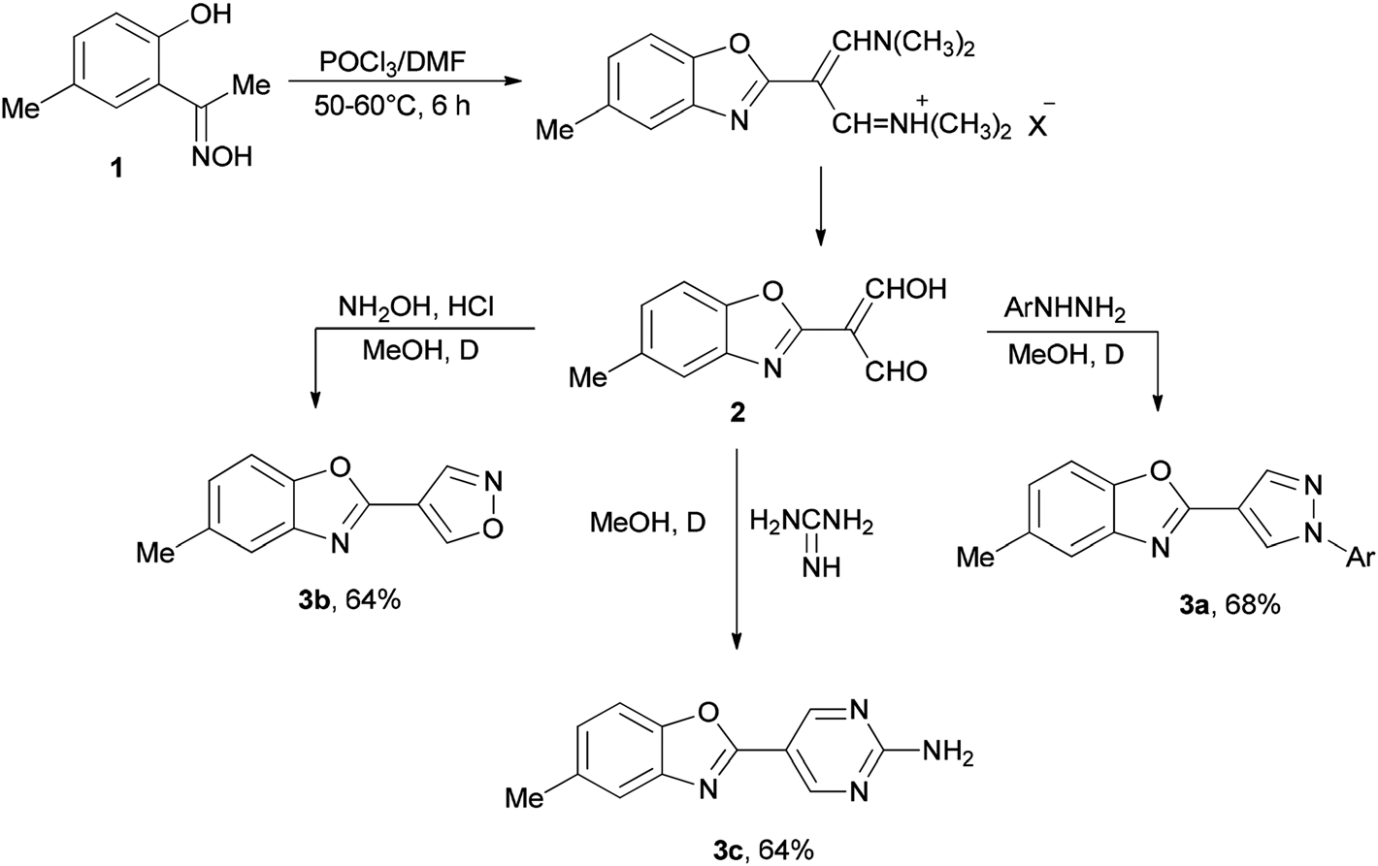
Synthesis of heterocyclic derivatives 3a–3c.

Initially, in 1973, Vilsmeier–Haack reagent (POCl_3_/DMF) was used for the formation of malondialdehyde 2 from 2-hydroxy-5-methylacetophenone oxime 1 through Beckmann rearrangement followed by cyclization. Kumar and co-workers in 2010 achieved and reported the synthesis of a series of novel heterocycles 3a–3c from compound 2 as a key intermediate (Scheme 1).^19^ The condensation reaction of 2 with suitable reagents afforded heterocyclic derivatives 3a–3c.^66^

**Scheme 2:**
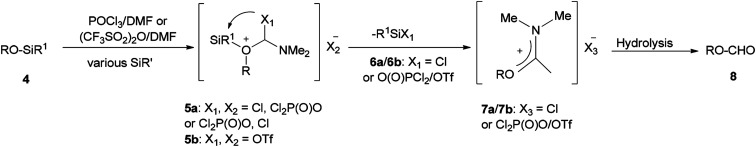
Preparation of formate esters 8 from *O*-TBDMS/*O*-TES protected alcohols 4.

In 1999, Lellouche and Koeller presented an efficient and convenient methodology for preparation of formate esters 8 from *O*-TBDMS/*O*-TES protected alcohols 4 (R alkyl residue) under Vilsmeier–Haack reaction conditions in a one-step fashion.^67^ A plausible mechanism is proposed and described in Scheme 2. In the first step; the POCl_3_/DMF complex ((CF_3_SO_2_)_2_O/DMF complex) is added to the silyl ether 4 to furnish the oxonium cation 5a (or 5b). In the second step; the formation of Si–Cl/Si–O strong bonds eliminates the R^1^Si–X_1_6a/6b (R^1^Si = TES or TBDMS) from the resulting intermediate 5a (or 5b) to produce *in situ* the relevant imidate salts 7a (or 7b) as a mixture of counter anions. The production of the desired formate esters 8 is completed by smooth hydrolysis of 7a (or 7b).

**Scheme 3:**
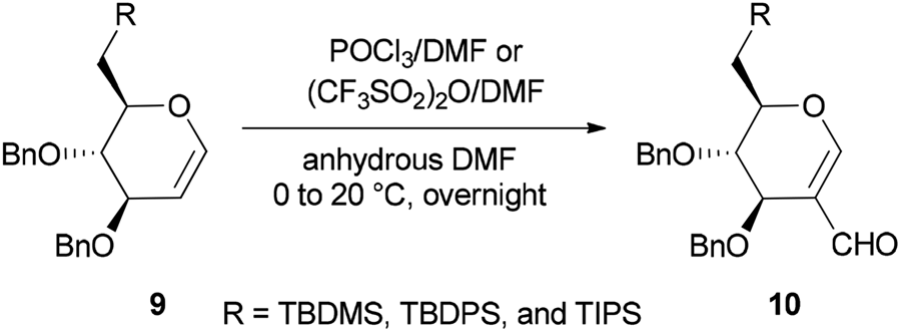
Preparing the corresponding C(6)-*O*-formates 10 from ethers 9.

Synthesis of the corresponding C(6)-*O*-formates 10 was accomplished and reported by the same group in 2001 through a one-step and selective transformation of *O*-TBDMS, *O*-TBDPS, and *O*-TIPS ethers of d-glucal 9, using two electrophilic Vilsmeier–Haack reagents POCl_3_/DMF or (CF_3_SO_2_)_2_O/DMF in anhydrous DMF at 0–20 °C (Scheme 3).^68^

**Scheme 4:**
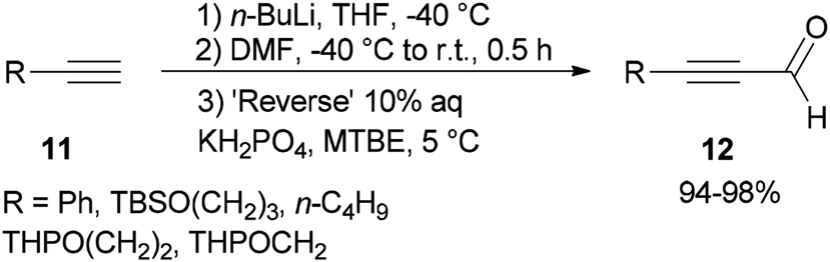
Synthesis of α,β-acetylenic aldehydes 12.

Synthesis of α,β-acetylenic aldehydes 12 was commenced with acetylides 11 which were initially converted to lithium acetylides in the presence of *n*-BuLi. The formylation of lithium acetylides was accomplished in the presence of DMF with subsequent quenching of the α-aminoalkoxide using 10% aqueous KH_2_PO_4_ to provide 12 (>94%) as sole products (Scheme 4).^69^

**Scheme 5:**
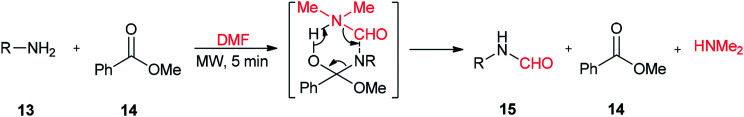
*N*-Formylation of amines.

A highly convenient and efficient method was developed by Jeon and Yang for *N*-formylation of various primary and secondary amines using DMF as a formylating agent in the presence of a catalytic amount of methyl benzoate as a promoter (Scheme 5).^70^ The main advantages of this methodology are short reaction time, high product yields, neutral reaction conditions, and selective *N*-formylation in the presence of a hydroxyl group.

**Scheme 6:**
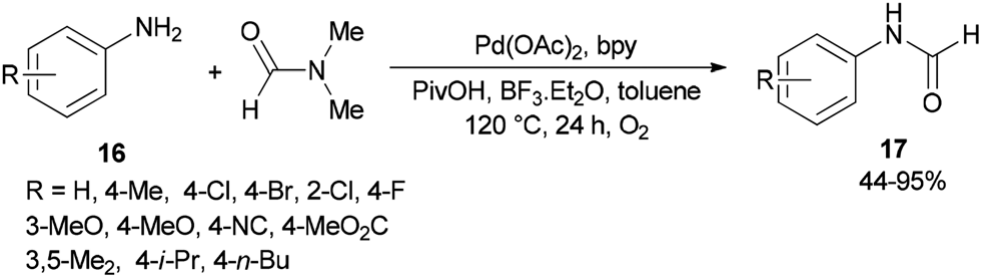
Transamidation of aniline derivatives 16 with dimethylformamide.

Gu and Guo developed a new protocol for efficient production of *N*-arylcarboxamides 17 through transamidation of aniline derivatives 16 with dimethylformamide in the presence of a catalytic amount of Pd(OAc)_2_, 2,2′-bipyridine, PivOH and BF_3_·Et_2_O as additives in toluene at 120 °C under O_2_ atmosphere (Scheme 6).^71^ This methodology opens a new way for the synthesis of various corresponding transamidation products from commercially available dimethylformamides and anilines.

**Scheme 7:**
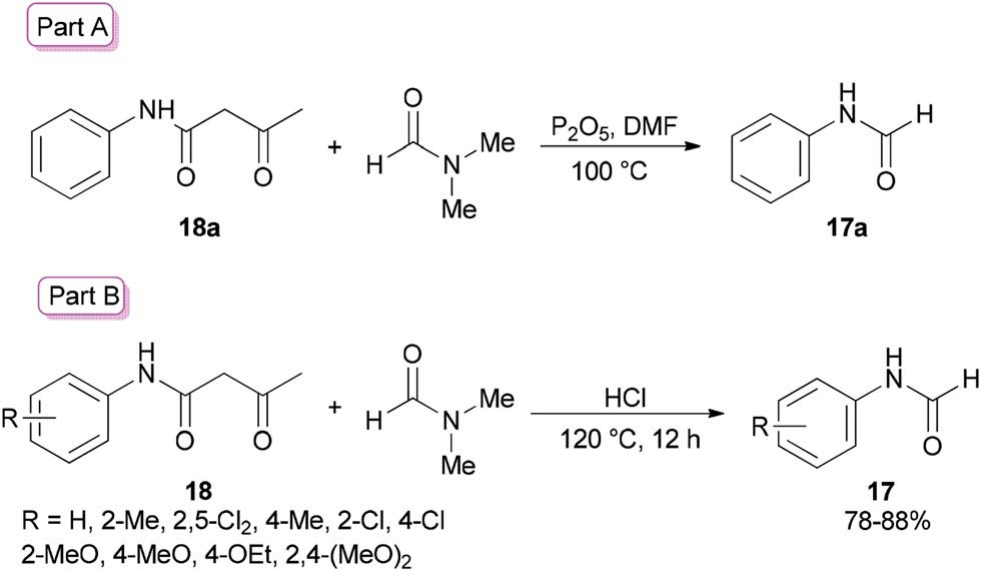
Synthesis of *N*-phenylformamides 17.

Amides and their derivatives as organic materials are very important in organic synthesis.^72,73^ They are widely used for preparing many medicines and biologically active compounds.^74,75^ A novel protocol, including the sequence of a carbon–nitrogen bond formation of β-keto amides with *N*,*N*-dimethylamides with subsequent two carbon–nitrogen bonds cleavage using HCl, was achieved by Chen and co-workers in 2015 for the construction of a wide range of R-benzyl formamides and acetamides as well as for the synthesis of various different acyl and aryl R-phenyl amide compounds.^76^*N*-Phenylformamide 17a was produced by reaction of acetoacetanilide 18a with DMF in the presence of P_2_O_5_ as the catalyst at 100 °C (Scheme 7, part A). Another novel protocol for the production of *N*-phenylamides 17 using dimethylformamide and dimethylacetamides 18 as the acyl donors was also developed. The acetoacetanilide derivatives 18 were reacted with DMF under optimal conditions (using HCl in DMF at 120 °C for 12 h) to furnish their corresponding *N*-phenylformamides 17 (Scheme 7, part B).

**Scheme 8:**
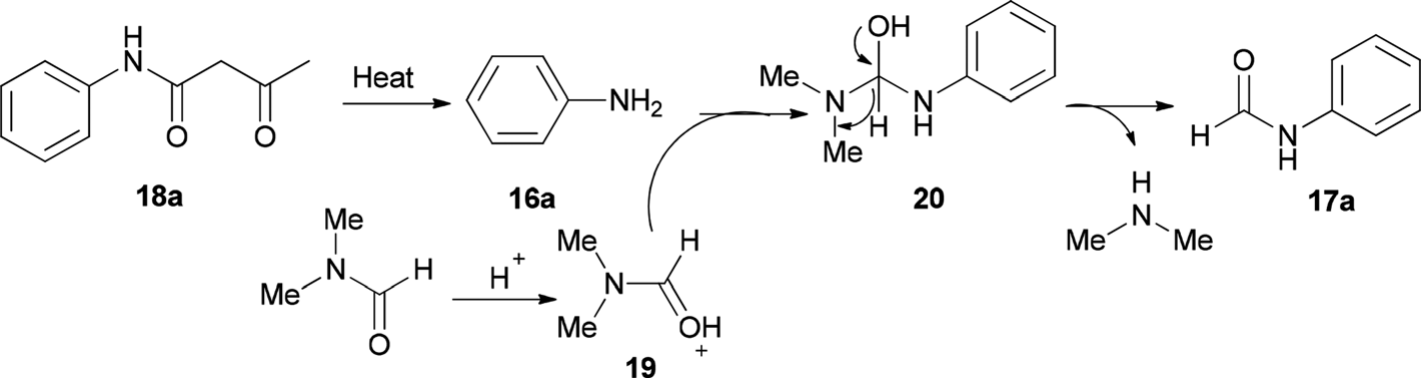
Rational mechanism for the synthesis of *N*-phenylformamides 17a.

As shown in Scheme 8, in the first step, the intermediates 19 and 16a were obtained by protonation of DMF and decomposition of 18a, respectively. Next, compound 20 was produced by nucleophilic attack of 16a on the carbonyl carbon leading to the product 17a by elimination of one equivalent of NHMe_2_ from 19.

Cyanoindoles are valuable intermediates in organic chemistry. They also are widely used in industry for the generation of pharmaceuticals, agrochemicals, and dyes.^77–82^ Because of their importance in organic synthesis, numerous methods for their formation have been developed.^83–89^

**Scheme 9:**
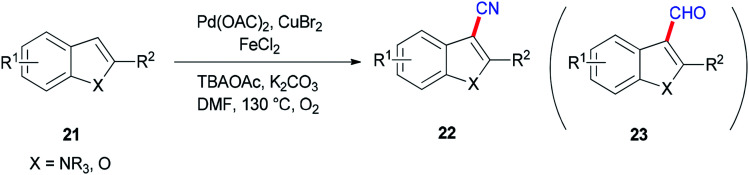
Generation of aryl nitriles 22.

Ding and Jiao in 2011 reported an alternative pathway to generate the aryl nitriles 22*via* new and direct Pd-catalyzed cyanation of indoles and benzofurans by C–H bond functionalization using DMF both as a source of CN and as a solvent (Scheme 9).^90^ Isotopic labeling experiments showed that nitrogen and carbon of the cyano group are generated from DMF.

**Scheme 10:**
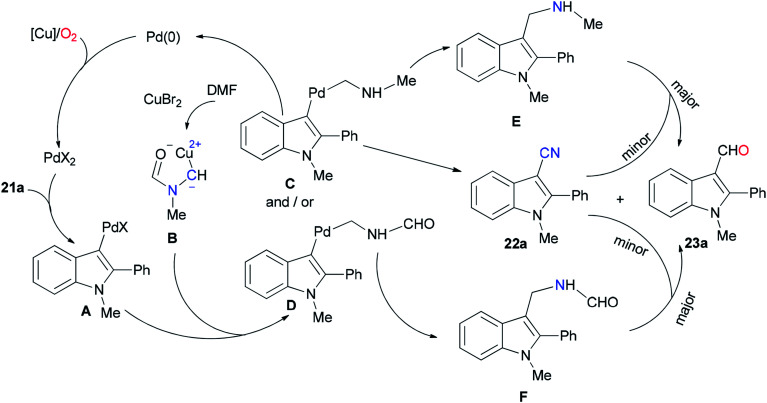
A probable mechanism for the generation of aryl nitriles 22.

As shown in Scheme 10, the Pd(0) intermediate A is formed by electrophilic aromatic palladation which undergoes an electrophilic reaction with B to give the intermediates C and/or D. The intermediates E and/or F are obtained by reductive elimination of C or D, respectively. The catalytic cycle is completed by reoxidization of the formed Pd(0) complex to Pd(ii) by employing O_2_ and/or Cu(ii) salts. Likely, the cyano product 22a is produced either directly from active C or through E as the intermediate, *in situ*. On the other hand, aldehyde 23a can be formed by oxidation of intermediate F.

**Scheme 11:**
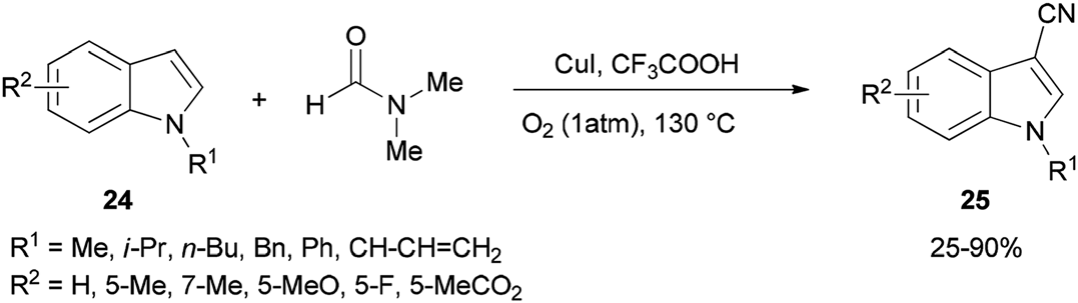
Synthesis of cyanoindoles 25 from indoles 24 and DMF.

Cheap and safe DMF as a CN source and as a solvent was applied in 2015 by Chen and Han for synthesis of the corresponding C3-cyanoindoles 25*via* direct and selective copper-mediated C3-cyanation of indole C–H bonds under an oxygen atmosphere (Scheme 11). A series of control experiments were performed to clarify the source of the ‘CN’ unit. There is no doubt that the nitrogen and carbon atoms come from DMF.^91^

**Scheme 12:**
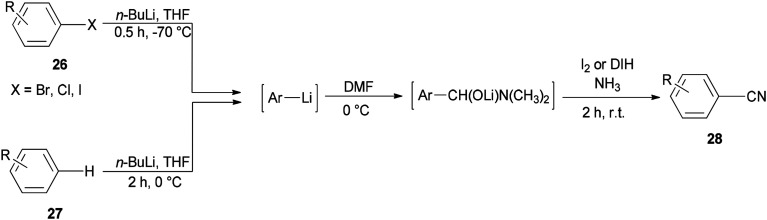
Generation of the aromatic nitriles 28.

Togo and co-workers generated the corresponding aromatic nitriles 28 by treatment of various aromatic halides 26 and various aromatic compounds 27 in the presence of *n*-BuLi and then DMF, followed by reaction with I_2_ in aq NH_3_ (Scheme 12).^92^

**Scheme 13:**
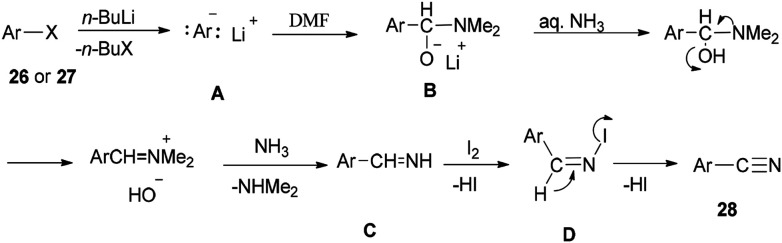
Plausible reaction mechanism for generation of the aromatic nitriles 28.

Scheme 13 shows a plausible reaction mechanism for production of the aromatic nitriles 28. First, aromatic halide 26, or any aromatic compound 27, is reacted with *n*-BuLi to afford aryllithium A. The second step is formation of the adduct B by treatment of A with DMF. *N*-Iodo aromatic imine D is yielded *via* addition of I_2_ or DIH (1,3-diiodo-5,5-dimethylhydantoin), and aq NH_3_, followed by reaction with I_2_, to afford the aromatic nitrile 28 through HI elimination.

**Scheme 14:**
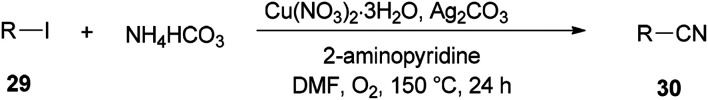
Cyanation of various aryl iodides 29.

Successfully, Chang and Pawar in 2014 employed DMF and ammonium bicarbonate as a combined source of “CN” units for the Cu(ii)-catalyzed cyanation of electron-rich and fused aryl iodides 29 (Scheme 14).^93^

**Scheme 15:**
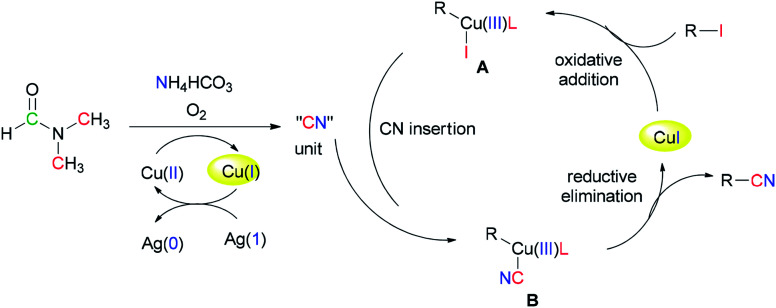
Proposed mechanism for catalytic cyanation.

A proposed mechanism for this catalytic cyanation is illustrated in Scheme 15. With respect to the key roles of copper(ii) species in the *in situ* production of “CN” units and subsequent cyanation of aryl halides, Ag_2_CO_3_ re-oxidizes the resultant copper(i) species under copper-mediated oxidative conditions.

**Scheme 16:**
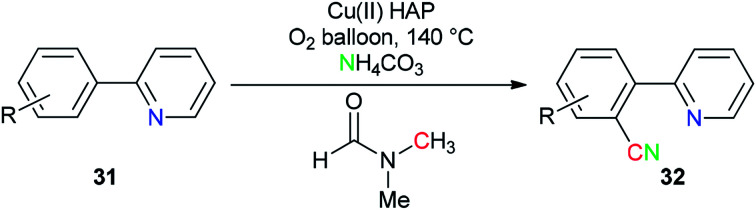
Synthesis of aryl nitrile 32 from hetero aryls 31.

Copper(ii) oxide supported on hydroxyapatite [HAP:Ca_5_(PO_4_)_3_(OH)] was used by Venugopal's group as a selective and active catalyst for generation of ‘CN’ units from NH_4_HCO_3_ and DMF for the synthesis of aromatic nitriles 32 by safe cyanation of C–H bonds of heteroaryl compounds 31 (Scheme 16).^94^

**Scheme 17:**
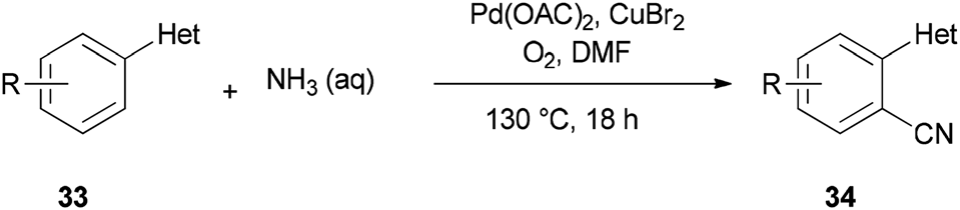
Synthesis of monosubstituted nitriles 34.

A unique pathway for synthesis of monosubstituted nitriles 34*via* regioselectivity cyanation at arene C–H bonds using DMF and NH_3_ as a combined source of “CN” unit was presented in 2010 by Kim and Chang (Scheme 17).^95^ Isotopic incorporation experiments indicated that the C and N of the “CN” are derived from the *N*,*N*-dimethyl moiety of DMF and NH_3_, respectively.

**Scheme 18:**
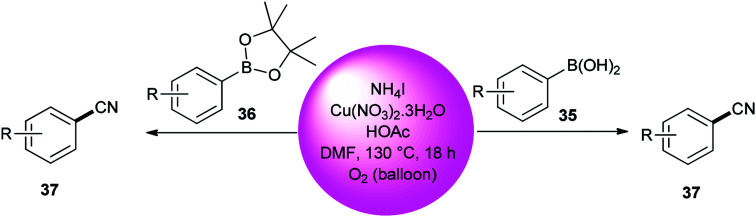
Cyanation of boronic acids 35 and aryl boronate esters 36.

Kim and co-workers used DMF (source of carbon atom) and NH_4_I (source of nitrogen and iodide atoms) in cyanation of aromatic boronic acids 35 and boronate esters 36 under Cu-mediated oxidative conditions (Scheme 18).^95^ The reaction is perceived to proceed in a two-step process: iodination and cyanation.

**Scheme 19:**
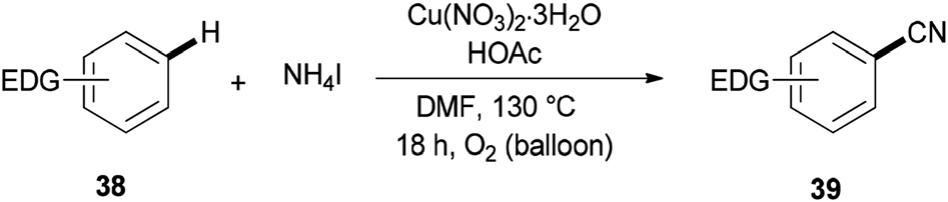
Cyanation of electron-rich arenes 38.

Cyanation of benzenes 38 bearing electron-rich groups on the phenyl ring would be probable because of their talent to perform the initial iodination step. Cyanation of arene C–H bonds was accomplished for the first time by using NH_4_I and DMF under oxidative copper-mediated conditions (Scheme 19).^95^

**Scheme 20:**
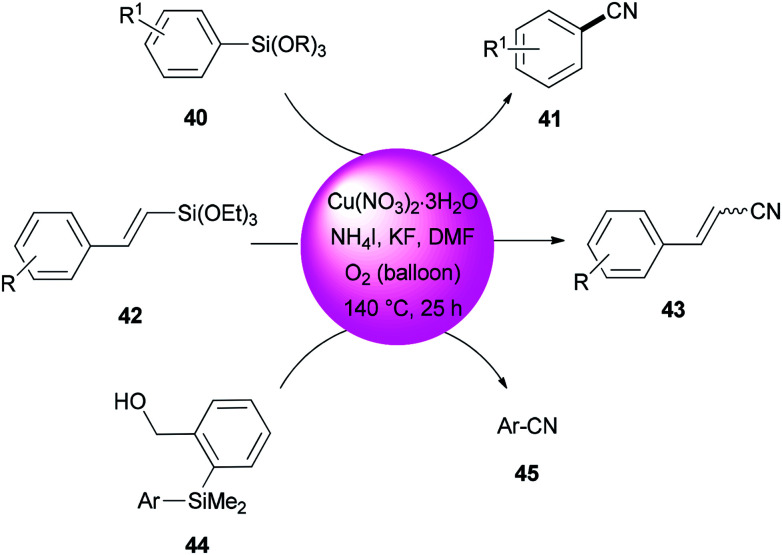
Cyanation of arylsilanes 40, vinylsilanes 42, and Hiyama silanes 44.

In 2013, DMF and iodide as a combined source of a “CN” unit were used by Wang and Chang for cyanation of organosilanes, such as arylsilanes 40, vinylsilanes 42, and Hiyama silanes 44 under copper-mediated oxidative conditions (Scheme 20).^96^ As a proposal, the reaction proceeds through two sequential steps: the initial transformation of organosilanes to their corresponding iodo intermediates followed by conventional cyanation.

**Scheme 21:**
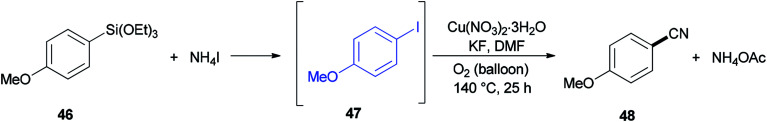
Reaction profile for cyanation of triethoxy(4-methoxyphenyl)silane 46.

A reaction profile for cyanation of triethoxy(4-methoxyphenyl)silane 46 was presented under the same reaction conditions (Scheme 21). It commenced with 46, which was transformed almost quantitatively to 4-methoxyiodobenzene 47. Then, with the disappearance of 47, formation of 4-methoxybenzonitrile 48 was started. As a matter of fact, when 48 was subjected to reaction conditions, 47 was provided in 74% yield even in the absence of potassium fluoride, proposing that iodoarenes act as a key intermediate in this cyanation process.

**Scheme 22:**
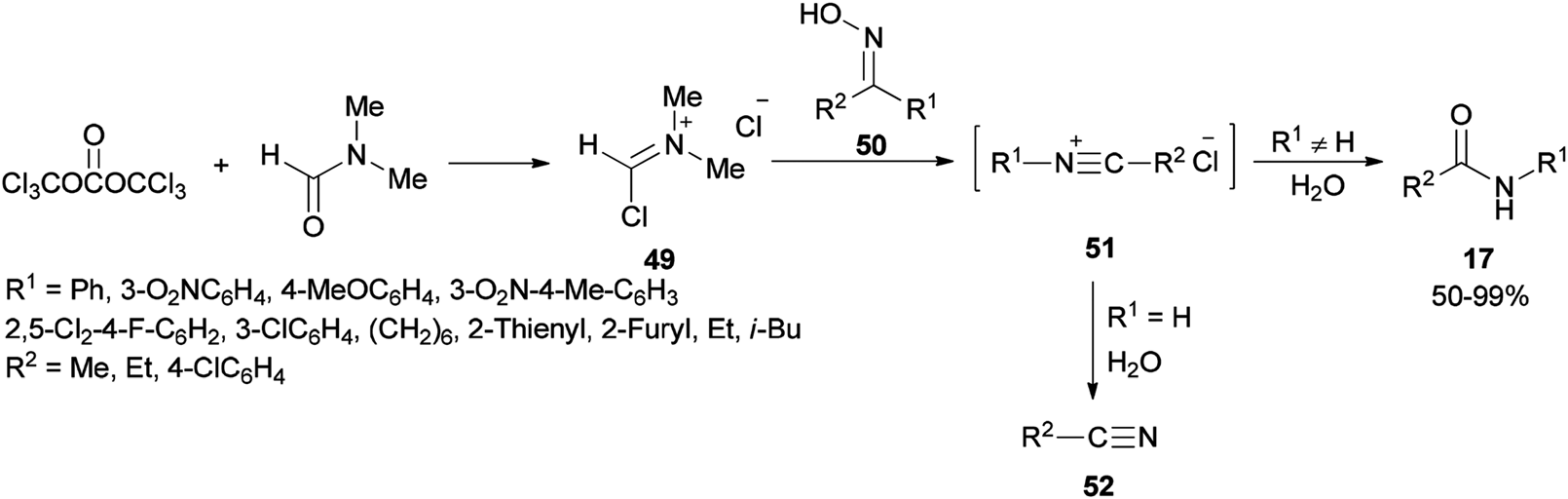
Synthesis of amides 17 and nitriles 52.

Preparation of amides from ketoximes is known as the Beckmann rearrangement which is generally carried out at high temperatures and in the presence of large amounts of strong acid. This method is applied both in organic chemistry and chemical manufacturing.^97–104^ Hence, development of a simple, and affordable catalytic system for the Beckmann rearrangement was desired. Su and co-workers synthesized amides 17*via* Beckmann rearrangement of oximes 50 using Vilsmeier salt reagent [Me_2_N^+^=CHCl] 49 which was produced from treatment of bis-(trichloromethyl) carbonate (BTC) with DMF in refluxing acetonitrile (Scheme 22).^105^ The fact that the reaction yield was reduced in the absence of DMF or an excess of DMF implies that without using DMF, no Vilsmeier reagent was formed and in excess of DMF, a less reactive adduct was formed. Su's group also synthesized nitriles 52 from intermediates 51.

**Scheme 23:**
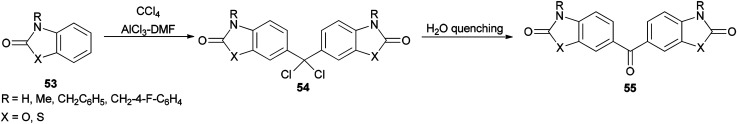
Synthesis of symmetrical benzophenones 55.

In 1997, Ucar and co-workers achieved and reported the synthesis of a series of symmetrical benzophenone derivatives 55 as single compounds by regioselective Friedel–Crafts C-alkylation reaction of 2(3*H*)-benzoxazolone and 2(3*H*)-benzothiazolone with carbon tetrachloride (CCl_4_) by use of AlCl_3_-DMF reagent (Scheme 23).^106^

**Scheme 24:**
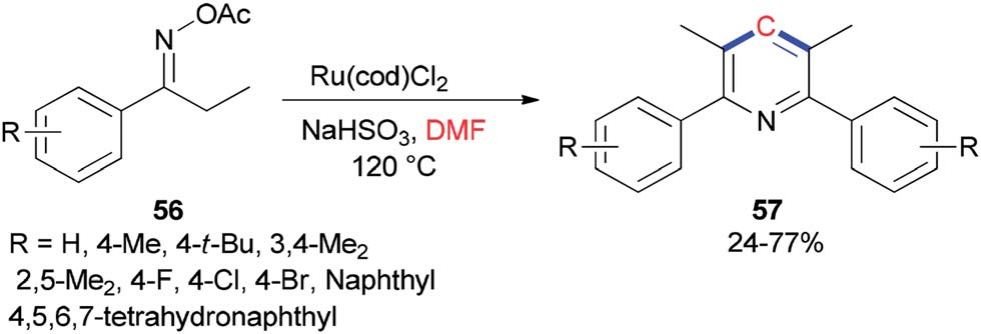
Ru-catalyzed cyclization of aryl ethyl ketoxime acetates 56 using DMF.

Guan and co-workers prepared tetra-substituted symmetrical pyridines 57*via* cyclization of ketoxime carboxylates 56 with *N*,*N*-dimethylformamide catalyzed by ruthenium under mild reaction conditions (Scheme 24).^107^ On the basis of deuterium-labeling experiments accomplished by Guan's group, the carbon unit is produced by a methyl carbon on DMF as a source of one carbon synthon.

**Scheme 25:**
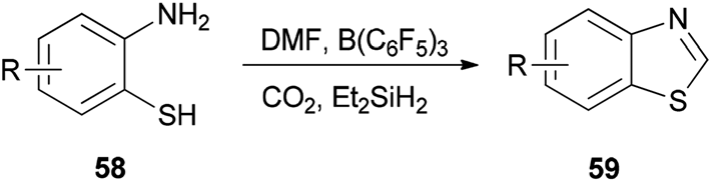
Synthesis of various benzothiazoles 59.

**Scheme 26:**
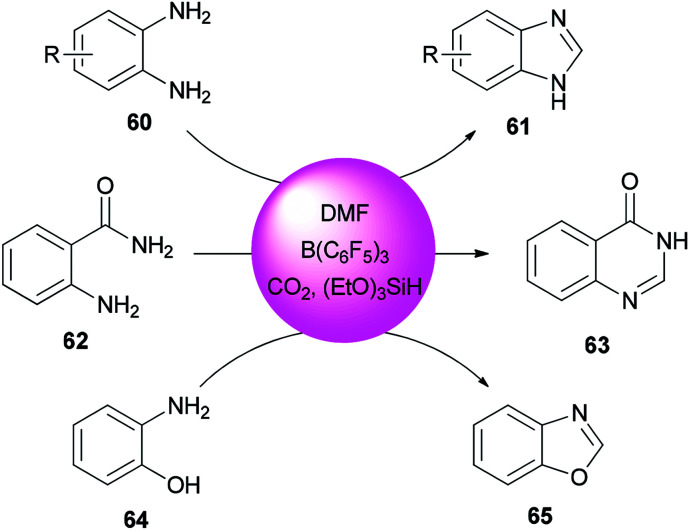
Synthesis of benzimidazoles 61, quinazolinone 63 and benzoxazole 65.

Heterocycles containing nitrogen are widely employed as pharmaceutical and agrochemical agents.^108–110^ They also extensively act as antibacterial, antiviral, antitubercular, anticancer, antihistaminic, anti-inflammatory, and antihypertensive agents as well as plant growth regulators.^111–116^ As a new metal-free catalytic system, generation of a series of heterocycles containing nitrogen including benzothiazoles 59, benzomidazoles 61, quinazolinone 63, and benzoxazole 65 were achieved and reported through cyclization of *ortho*-substituted aniline derivatives with DMF using B(C_6_F_5_)_3_ along with CO_2_ (Schemes 25 and 26).^117^

**Scheme 27:**
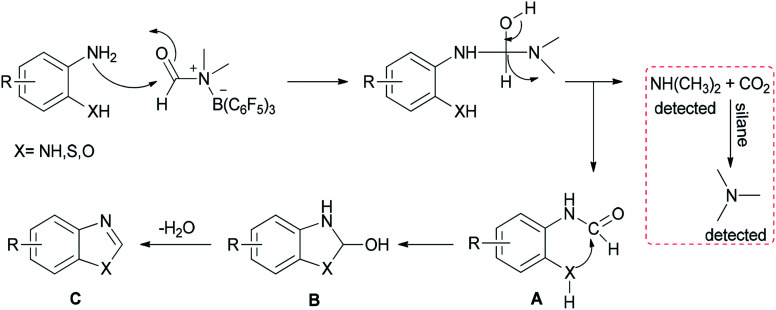
Mechanistic proposal for the synthesis of heterocycles containing nitrogen.

A mechanistic proposal for this reaction is outlined in Scheme 27. First, B(C_6_F_5_)_3_ activates DMF through electrostatic interaction. Next, formylated intermediate A provided by nucleophilic attack of substrates undergoes intramolecular nucleophilic cyclization and loss of H_2_O to yield products C. Simultaneously, released dimethylamine is treated with CO_2_ and silane to give the trimethylamine product which pushes the reaction to the right; as a result, the reaction is greatly promoted to proceed to completion.

**Scheme 28:**
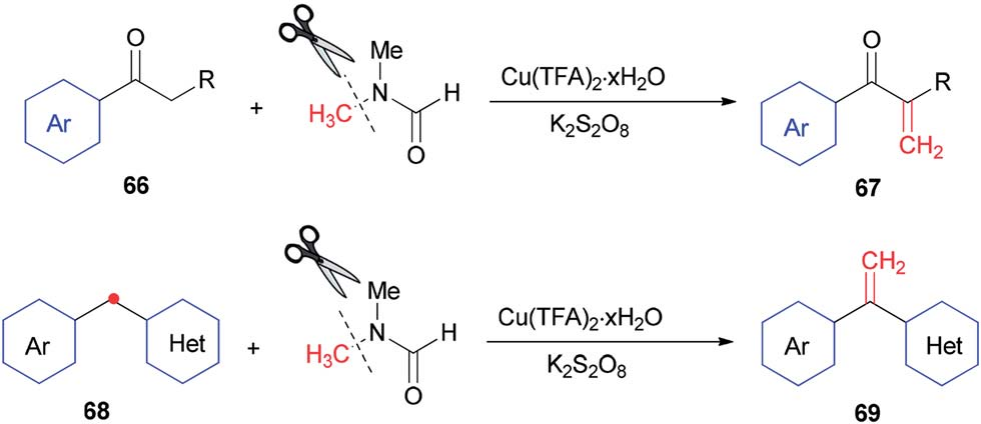
α-Methylenation of arylketones 66 and arylvinylpyridines 68.

In 2014, Lei and co-workers reported a simple method for the synthesis of terminal olefins including arylvinylketones 67 and arylvinylpyridines 69 by Cu-catalyzed direct oxidative Csp^3^–H methylenation of arylketones 66 and 1-aryl-1-pyridinemethanes 68, respectively, using DMF as one the sources of carbon and as a solvent (Scheme 28).^118^ Investigation of a preliminary mechanism shows that CH_2_ comes from DMF (N–CH_3_).

**Scheme 29:**
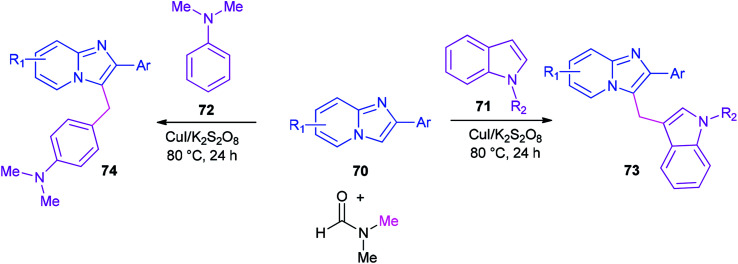
Synthesis of heterodiarylmethanes 73 and 74.

**Scheme 30:**
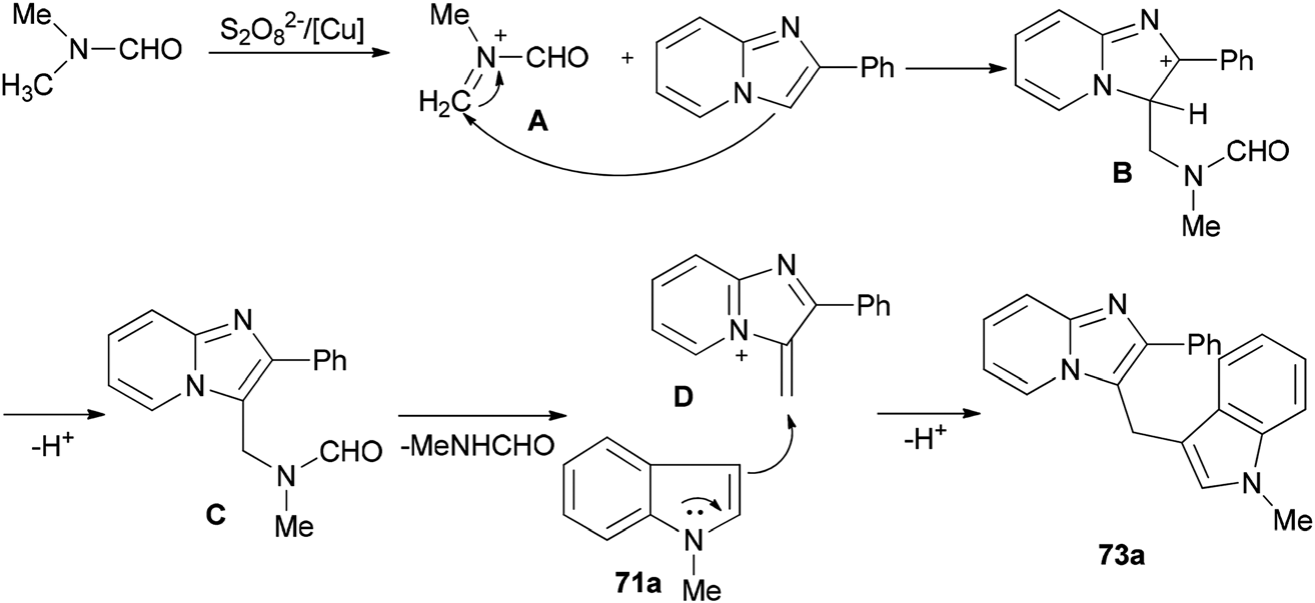
A possible mechanism for the synthesis of heterodiarylmethane 73a.

Syntheses of heterodiarylmethanes 73 and 74 was achieved by Hajra and co-workers in 2016 through a copper-catalyzed coupling reaction of imidazo[1,2-*a*]pyridines 70 using DMF as a methylenating reagent (Scheme 29).^119^ A series of 3-(1*H*-indol-3-ylmethyl)-imidazo[1,2-*a*]pyridine derivatives 73 were prepared under aerobic reaction conditions. This method is also appropriate to prepare (4-imidazo[1,2-*a*]pyridin-3-ylmethyl)(phenyl)dimethylamines 74. The current methodology opens a new way for synthesis of important building blocks of the heterodiarylmethanes. A possible mechanism for this reaction was proposed (Scheme 30).^119^

**Scheme 31:**
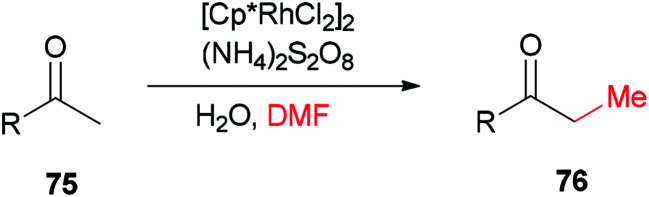
Rh-catalyzed direct methylation of ketones 75 with DMF.

Xue and co-workers in 2014 presented a highly effective protocol which allows rhodium-catalyzed direct methylation of ketones 75 using DMF (Scheme 31).^120^ Mechanistic studies indicate that the DMF plays dual roles: as the source of carbon for methylation and source of hydrogen for the rhodium-catalyzed reduction of the methylene into a methyl group.

**Scheme 32:**
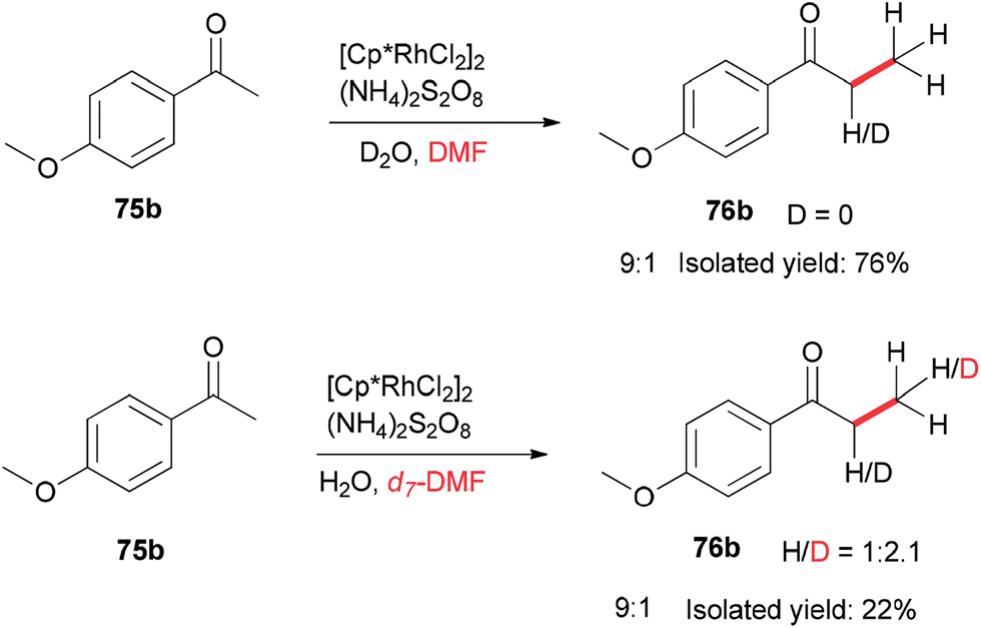
Investigation of the reaction mechanism for Rh-catalyzed methylation of ketone 75b.

To gain insight into the reaction mechanism, deuterium labeling experiments were accomplished. Replacing H_2_O with D_2_O did not significantly affect the product yield. When DMF was exchanged to *d*_7_-DMF, the reaction became significantly slower. Thus, it is suggested that the newly formed methyl group is derived from DMF due to a *H*/*D* ratio of 1 : 2.1 observed on the newly assembled methyl group (Scheme 32).

**Scheme 33:**
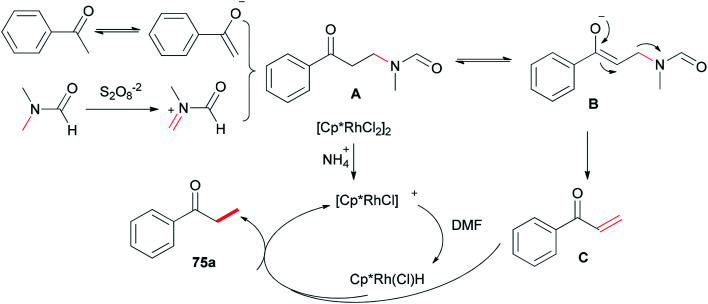
A probable mechanism for the Rh-catalyzed methylation of ketone 75b.

A probable mechanism was proposed which is illustrated in Scheme 33. Persulfate oxidizes DMF to give an iminium intermediate. The intermediate A generated by attack of enolate is converted to intermediate B followed by C–N bond cleavage to create unsaturated ketone intermediate C. Next, the latter is reduced through a Rh–H complex, which is probably created from dehydrogenation of DMF by [Cp*RhCl_2_]_2_, leading to the methylated product 75a, with the transfer process a methyl group from DMF to the ketone. In the reduction step, (NH_4_)_2_S_2_O_8_ can help the dissociation of Cl from Rh(iii) through hydrogen bonding with its NH_4_^+^ ion.

**Scheme 34:**
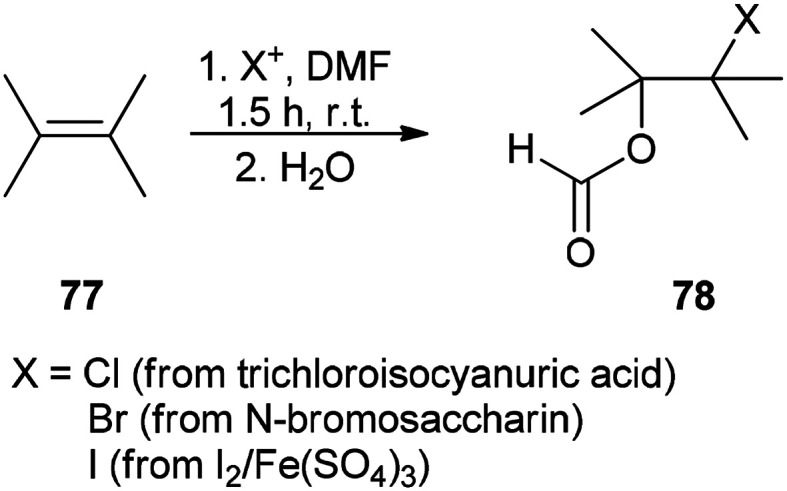
Generation of the corresponding β-haloformate 78 by treatment of alkenes 77 with DMF.

The corresponding β-haloformates 78 were generated in high regio- and stereoselectivity by treatment of alkenes 77 with DMF in the presence of sources of electrophilic halogens (trichloroisocyanuric acid or *N*-bromosaccharin or I_2_/Fe_2_(SO_4_)_3_) followed by addition of water (Scheme 34).^121^

**Scheme 35:**
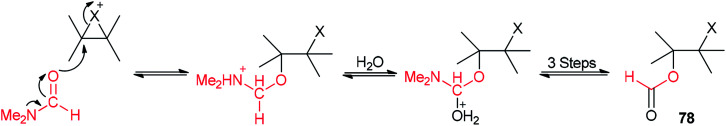
A mechanism for synthesis of the corresponding β-haloformate 78.

A mechanistic scheme for this transformation is shown in Scheme 35. The halonium ion intermediate is converted to an iminium ion by nucleophilic attack of the oxygen of DMF. Then, the hydrolysis of iminium ion yields the β-haloformate.

**Scheme 36:**
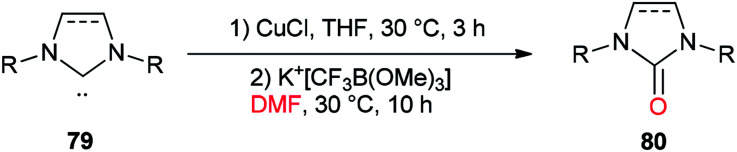
Formation of complicated imidazolinones 80.

In 2013, a unique and efficient method was reported by Chen and co-workers for the formation of complicated imidazolinones 80 from carbene complexes 79*via* an oxygen-atom insertion reaction of NHC-copper complexes using DMF as the source of oxygen (Scheme 36).^122^

**Scheme 37:**
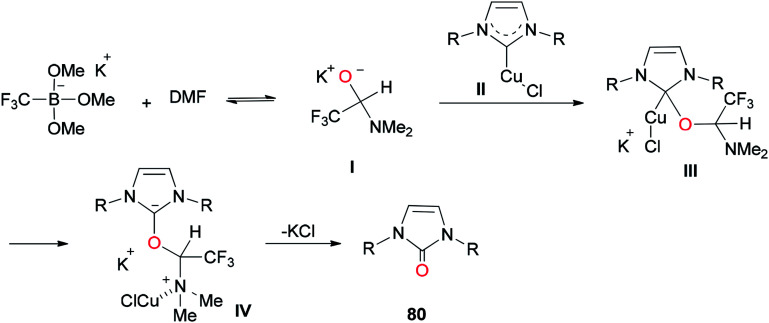
A mechanistic scheme for the formation of complicated imidazolinones 80.

A mechanistic scheme for the formation of complicated imidazolinones 80 is presented in Scheme 37. The negative oxygen ion intermediate I generated by reaction of the trifluoromethyl borate salt with DMF is transformed to less stable complex III through oxygen-atom insertion of NHC–copper complex II. Next, the copper chloride is dissociated and the carbanionic intermediate IV is produced in a similar path to Zhou's mechanism.^123,124^ At the late stage, IV undergoes a C–N bond cleavage, leading to the desired product 80.

**Scheme 38:**
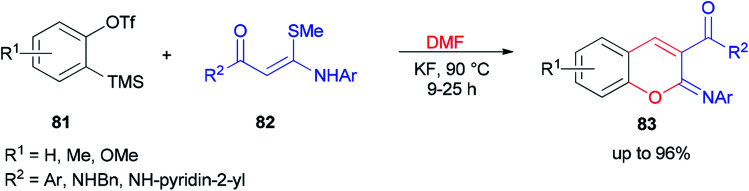
Synthesis of 2-aryliminochromenes 83.

After a couple of years, Li and co-workers synthesized 2-aryliminochromenes 83 by a transition-metal-free reaction between arynes, *N*,*S*-keteneacetals 82 and dimethylformamide, where dimethylformamide acts as a source of oxygen to form chromeneskeleton 83 (Scheme 38).^125^

**Scheme 39:**
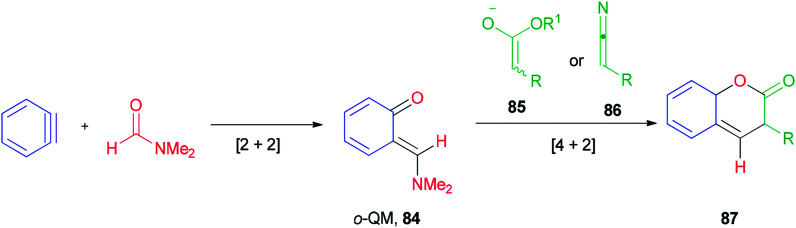
Production of coumarins 87.

*Ortho*-Quinone methide 84 produced by [2 + 2] cycloaddition of aryne with DMF was efficaciously coupled with ester enolates or ketenimine anions under a [4 + 2] cycloaddition to provide direct access to various coumarins 87, which included integral and key parts of active biological compounds and pharmaceuticals (Scheme 39).^126^

**Scheme 40:**
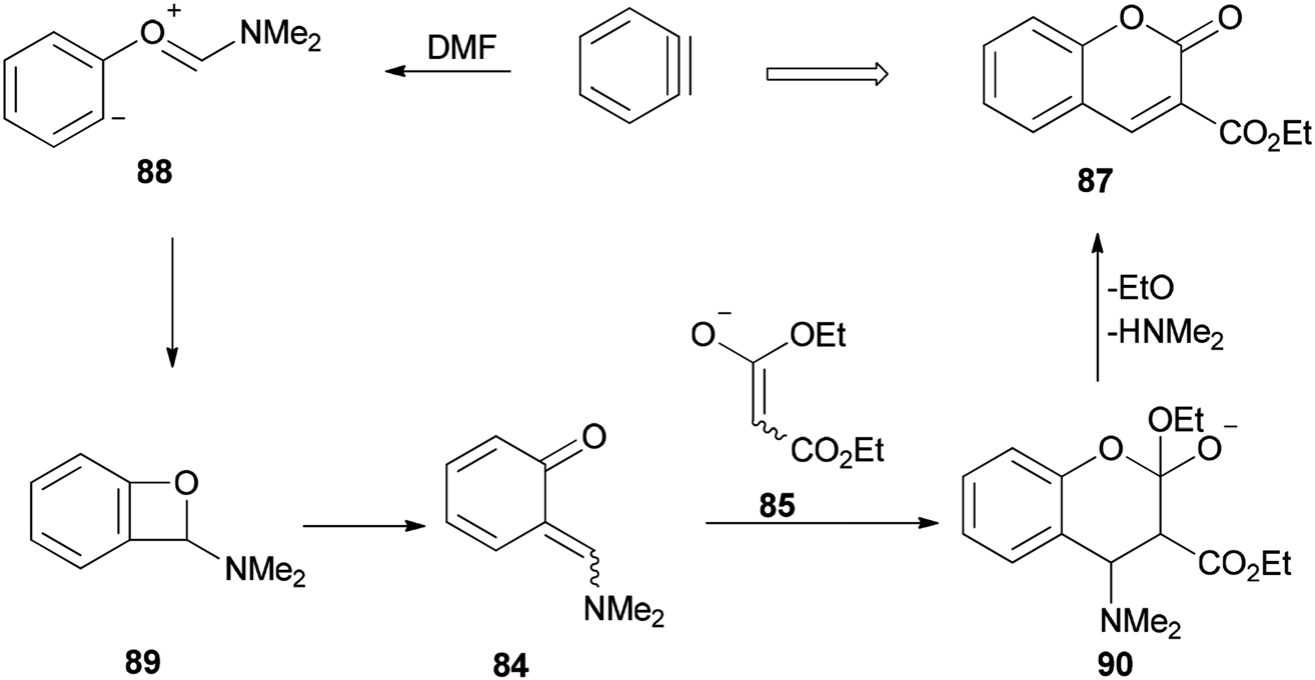
A mechanism for the production of coumarins 87.

The mechanism of this reaction is depicted in Scheme 40 and begins with a nucleophilic attack of oxygen of DMF to benzyne to afford the resulting zwitterion (88)^127^ which is subjected to intramolecular cyclization to furnish benzoxete 89. Then, an *ortho*-quinone methide 84 is obtained from 89 by electrocyclic ring-opening [4 + 2]. Cycloaddition of 84 with an enolate of 85, and then by elimination of a dimethylamine and ethoxide, gives 87.

**Scheme 41:**
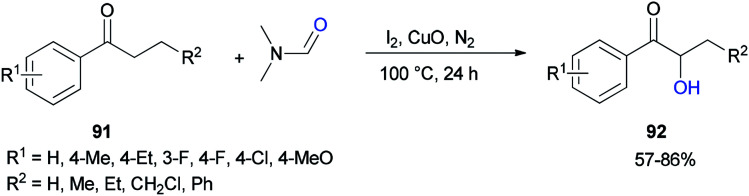
Synthesis of α-hydroxy arones 92 from α-substituted arones 91.

Liu and co-workers in 2017 used DMF as a source of oxygen for the production of α-hydroxy arones 92. Treatment of propiophenones 91 with DMF *via* α-hydroxylation reaction in the presence of iodine, copper oxide, and N_2_ afforded the corresponding α-hydroxylated products 92 (Scheme 41).^128^ The results demonstrated that the electronic effects of the substituents on the phenyl ring played a key role in this reaction. Among different copper species, CuO was found to be the most efficient oxidant.

**Scheme 42:**
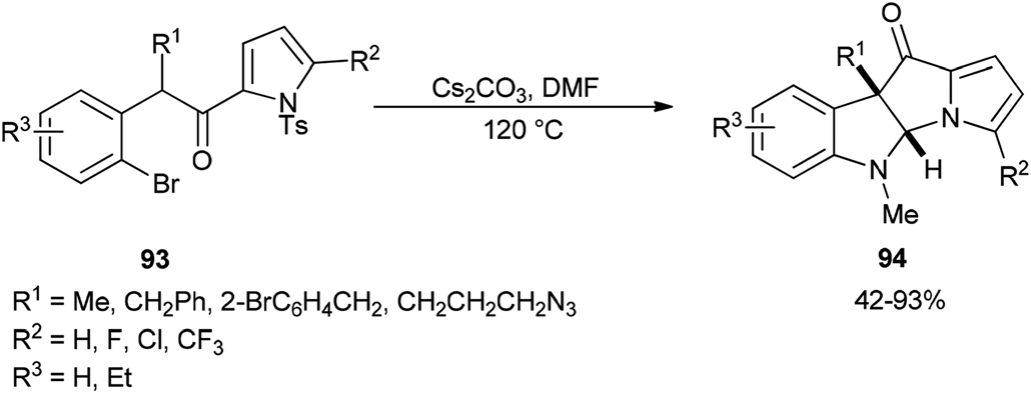
Formation of dihydropyrrolizino[3,2-*b*]indol-10-one 94.

The formation of dihydropyrrolizino[3,2-*b*]indol-10-ones 94 was accomplished *via* a Cs_2_CO_3_-promoted cascade reaction of *N*-tosyl-2-(2-bromophenylacetyl)pyrroles 93 with DMF in absence of any exogenous transition metal catalyst or ligand (Scheme 42).^129^

**Scheme 43:**
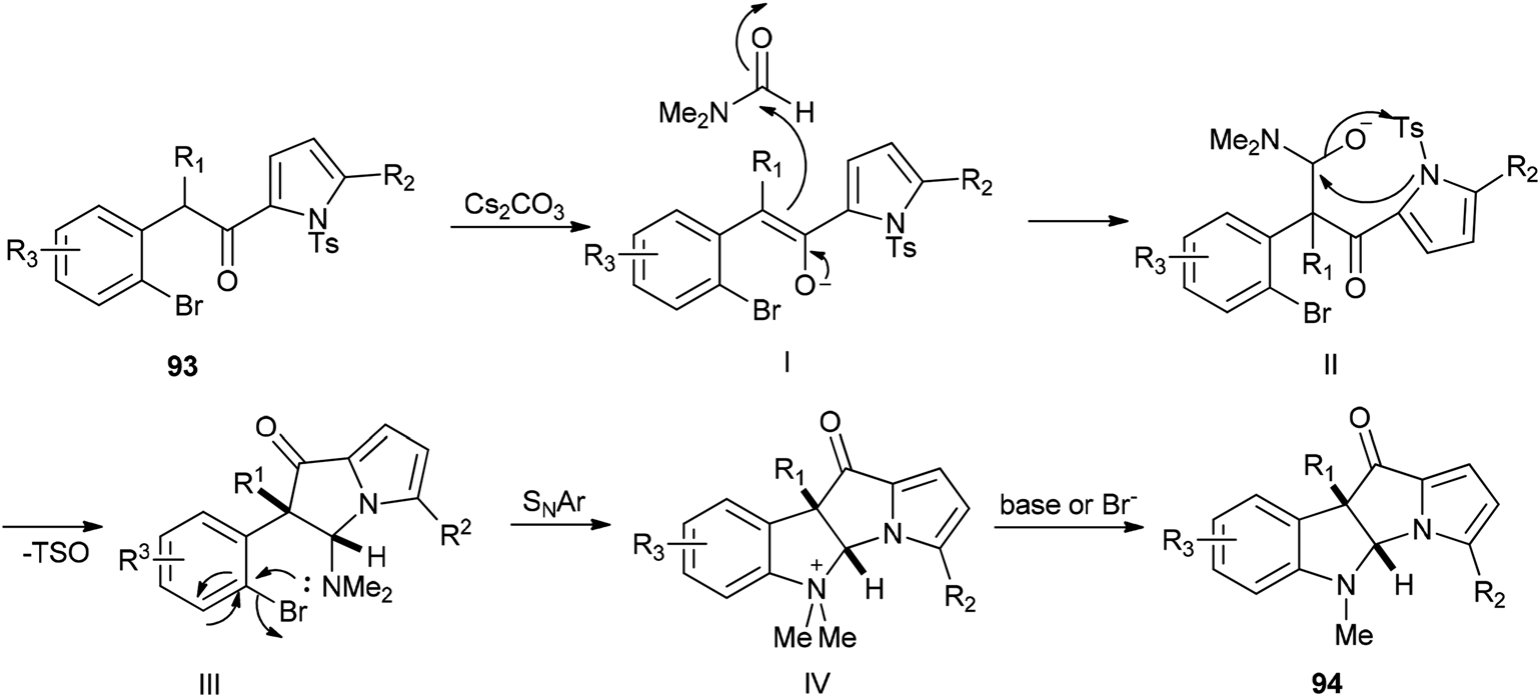
Proposed mechanism for the formation of dihydropyrrolizino[3,2-*b*]indol-10-one 94.

A plausible mechanism for this transformation is suggested through nucleophilic attack of DMF with enolate and pyrrolyl nitrogen followed by formation of C–N bond (Scheme 43).

**Scheme 44:**
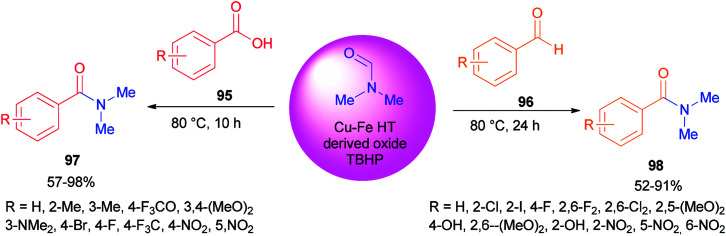
Amidation of carboxylic acids 95 and substituted benzaldehydes 96 with DMF.

Recently, carboxylic acids 95 and substituted benzaldehydes 96 were amidated with DMF as a source of NMe_2_ in the presence of TBHP as an oxidant and Cu–Fe hydrotalcite-derived (HT-derived) as a catalyst by Priya and co-workers (Scheme 44).^130^ Cu–Fe HT-derived oxide which is highly active, is prepared from hydrotalcite-like materials formed by co-precipitation of Cu and Fe followed by calcination.

**Scheme 45:**
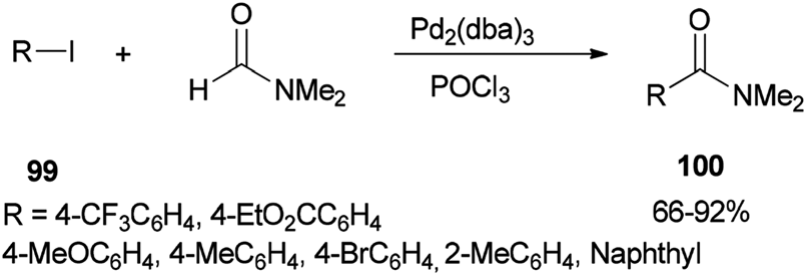
Synthesis of arylcarboxamides 100 from aryl halides 99 and *N*,*N* dimethylformamide.

In 2002, Nozaki and co-workers presented a novel and brief strategy to synthesize arylcarboxamides 100 from aryl halides 99 and *N*,*N*-dimethylformamide both as an amide source in one step and as a solvent without the need for using carbon monoxide 100 (Scheme 45).^131^

**Scheme 46:**
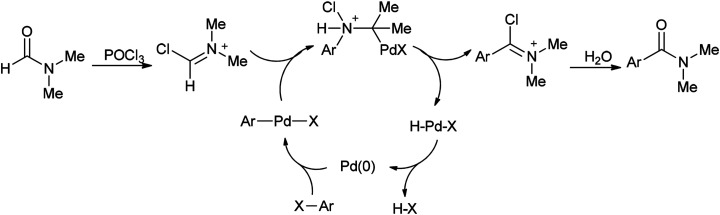
Proposed mechanism for the synthesis of arylcarboxamides 100.

A mechanism for the synthesis of arylcarboxamides 100 has been proposed. Initially, Heck-type addition of aryl halide to C–N double bond in iminium species [Me_2_N^+^=CHCl][Cl_2_P(=O)O^−^] occurs which is followed by β-hydride elimination. It also is likely this reaction take place through oxidative addition of aryl halide to Pd(0) under reaction conditions to yield the arylpalladium halide (Scheme 46).^132^

**Scheme 47:**
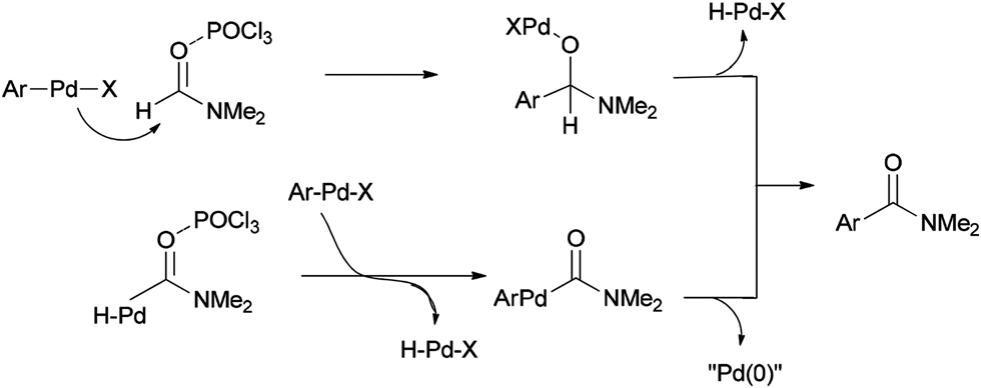
Two other proposed mechanisms for the synthesis of arylcarboxamides 100.

Two other proposed mechanisms are shown in Scheme 47. In the second mechanism, an amide product was produced through nucleophilic addition of arylpalladium halide to carbonyl activated with POCl_3_ (a Lewis acid) followed by β-hydride elimination. In the third mechanism, the reaction can be performed through oxidative addition of C–H bond of *N*,*N*-dimethylformamide and then by an exchange process and reductive elimination. The insertion of Ar–X to Ar-Pd-CONMe_2_, instead of the exchange furnished Pd(iv) may be an alternative and key precursor for the reductive elimination of product. It is important to note that in these three proposed mechanisms no base was used although in conventional examples, presence of a base for the regeneration of Pd(0) species from H–Pd–X is required.

**Scheme 48:**
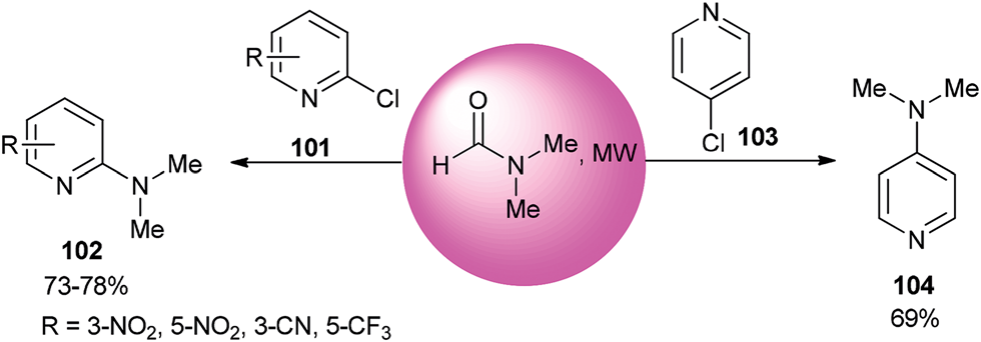
Reaction of 2- or 4-chloropyridine with DMF.

The reaction of 2- or 4-chloropyridine with DMF as an amine source and solvent under refluxing conditions afforded aminopyridines (Scheme 48).^133^

**Scheme 49:**
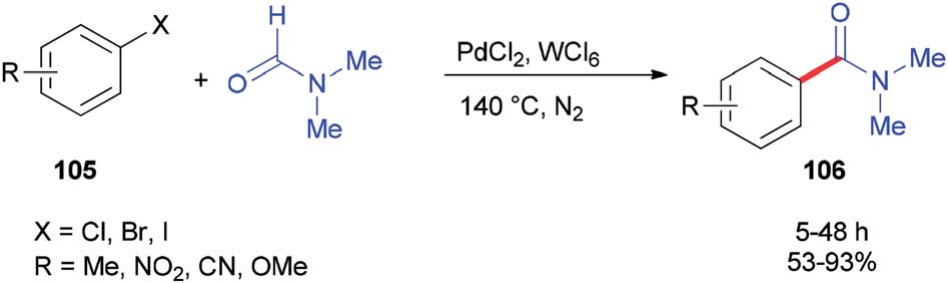
Synthesis of corresponding *N*,*N*-dimethyl amides 106 from aryl halides 105.

In 2014, Iranpoor and co-workers used WCl_6_/DMF as a Vilsmeier iminium type intermediate and a reducing system for generation of Pd(0) in aminocarbonylation of aryl halides 105. In this reaction, the corresponding *N*,*N*-dimethyl amides 106 were synthesized *via* treatment of aryl halides 105 (iodides, bromides, as well as chlorides) with *N*,*N*-dimethylformamide, using PdCl_2_ as a pre-catalyst without any phosphorous ligand under standard conditions (Scheme 49).^134^ Among metal halides, tungsten hexachloride (WCl_6_) was the most efficient. Reasonably, the Pd(ii) was easily reduced to Pd(0) using WCl_6_/DMF in comparison with other metal halides (MoCl_5_, ZrOCl_2_, ZrCl_4_, TiCl_4_, or FeCl_3_) in DMF.

**Scheme 50:**
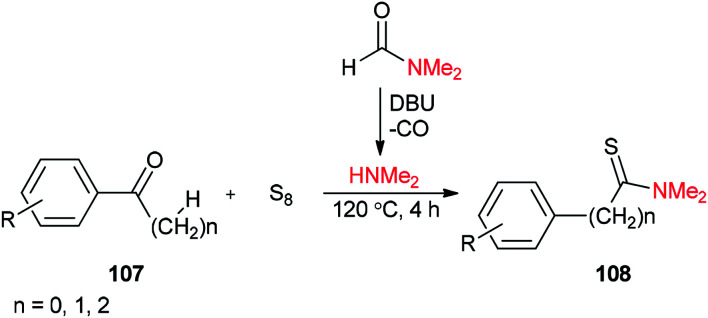
Synthesis of thioamides 108 using DMF.

Thioamides 108 were produced in 2015 by Liu and co-workers from compounds 107, octasulfur and dimethylamine which was prepared *in situ* from DMF through a base-induced cleavage (Scheme 50).^135^

**Scheme 51:**
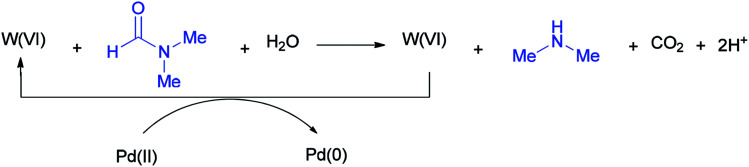
Conversion of W(vi) to W(iv) using DMF.

According to literature results, DMF acts as a reducing agent for the reduction of Ag(i) to Ag(0). On this basis, DMF can act as a reducing agent for the transformation of W(vi) to W(iv) which in turn acts as a reducing agent for the conversion of Pd(ii) to Pd(0) (Scheme 51).^136^

**Scheme 52:**
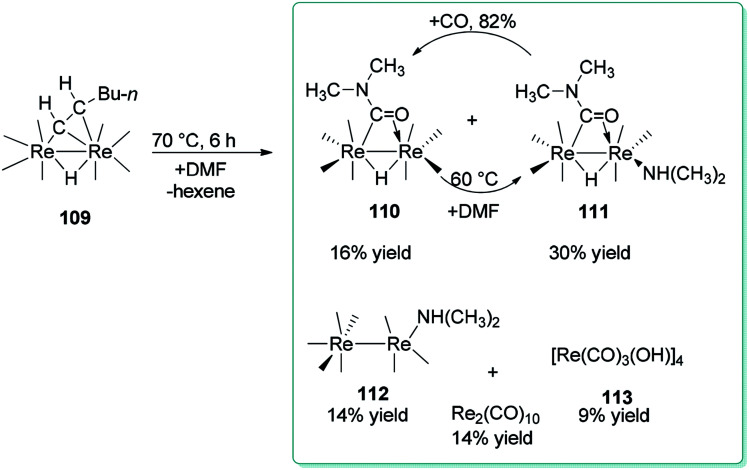
Products obtained from the reaction of 109 with DMF.

For the first time, Adams and Dhull reported the binuclear oxidation addition of a formyl C–H bond of DMF to produce a η^2^-bridging formamido (O=CNMe_2_) ligand with the C atom bonded to one rhenium (Re) atom and the O atom bonded to the other rhenium atom (Scheme 52).^137^ Elimination of hexane under heat in the presence of DMF led to three novel dirhenium products 110–112. Generation of small amounts of [Re(CO)_3_(OH)]_4_113, HRe(CO)_5_, and Re_2_(CO)_10_ were the result of reactions of 109 and 110 with traces of H_2_O in the DMF reagent.^138^

**Scheme 53:**
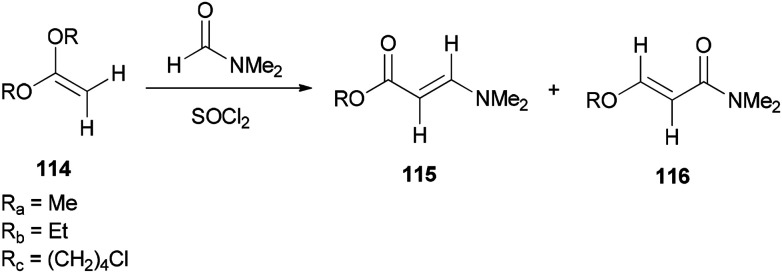
Generation of alkyl (*E*)-3-(dimethylamino)acrylates 115.

Bates's group presented a one-step method for the generation of alkyl (*E*)-3-(dimethylamino)acrylates 115 by reaction of ketene acetals 114 with DMF (Scheme 53).^139^

**Scheme 54:**
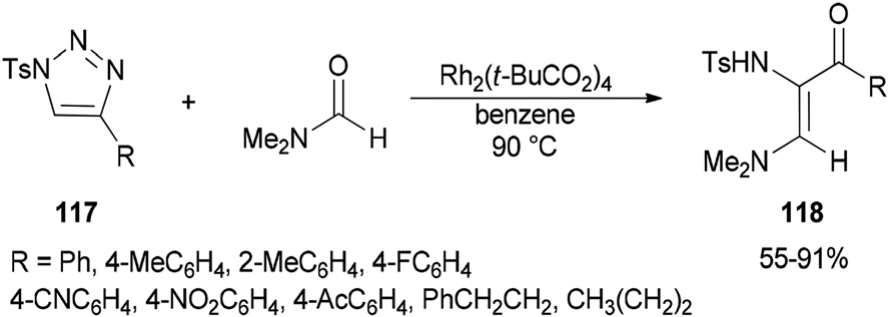
Formation of *cis*-diamino enones 118 from *N*-sulfonylated 1,2,3-triazoles 117.

Incorporation of all the atoms of a DMF molecule into the product was achieved by a formal stereoselective 1,2-insertion of rhodium(ii) azavinyl carbenes, produced *in situ* from *N*-sulfonylated 1,2,3-triazoles 117 using Rh_2_(*t*-BuCO_2_)_4_, into the C=O double bond of DMF to provide *cis*-diamino enones 118 (Scheme 54).^140^

**Scheme 55:**
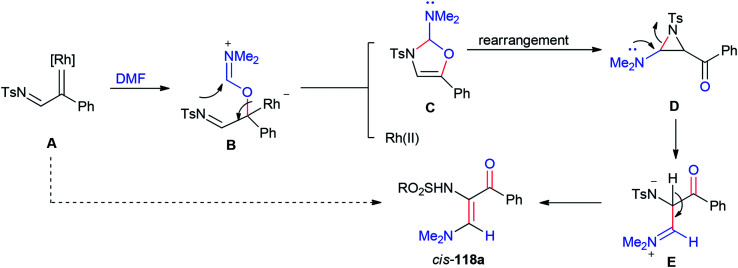
A plausible mechanism for the formation of 118a.

A plausible mechanism for the formation of 118a is illustrated in Scheme 55. Rhodium–iminium zwitterionic B prepared by stereoselective 1,2-insertion of A into the C=O bond of DMF resulted in the formation of 2-amino-4-oxazoline C as a product. The latter is rearranged to give aziridine D. At last, the synthesis of stable vinylogous enamino ketone 118a is completed by conversion of intermediate D by ring-opening and deprotonation.

**Scheme 56:**
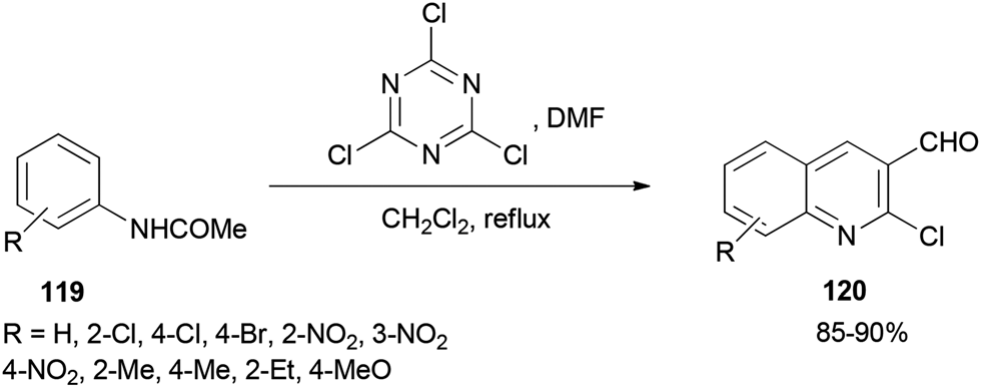
Synthesis of 2-chloroquinoline-3-carbaldehydes 120 by cyclization of acetanilides 119.

2-Chloroquinoline-3-carbaldehydes 120 were synthesized by Rajanna and co-workers through cyclization of acetanilides 119 with 2,4,6-trichloro-1,3,5-triazine/*N*,*N*-dimethylformamide (TCTA/DMF) adduct as a Vilsmeier–Haack type reagent under conventional and ultrasonically assisted reaction conditions (Scheme 56).^141^ The reaction time under sonication is reduced in comparison to when reactions are performed under conventional reaction conditions.

**Scheme 57:**
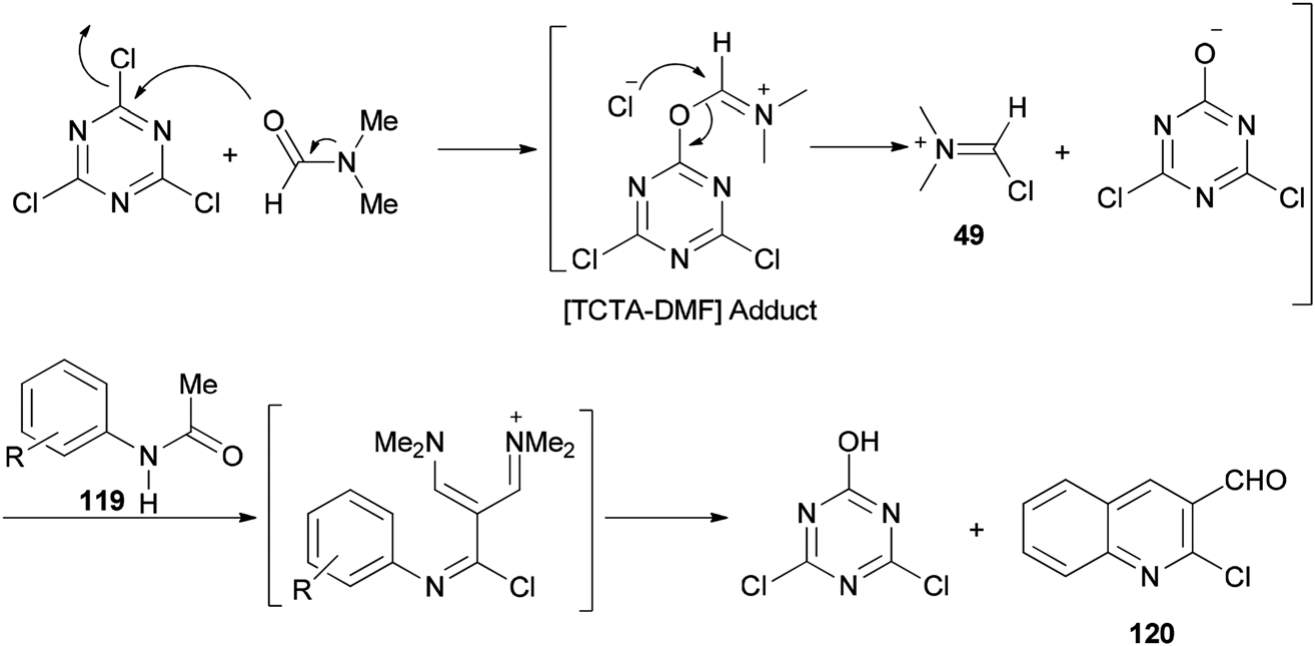
A mechanistic proposal for the synthesis of 2-chloroquinoline-3-carbaldehydes 120.

The mechanism starts with preparing [TCTA–DMF] adduct which is converted to (chloromethylene)dimethyliminium. The reaction of the cationic intermediate with acetanilide 119 furnished the 2-chloroquinoline-3-carbaldehydes 120 (Scheme 57).

**Scheme 58:**
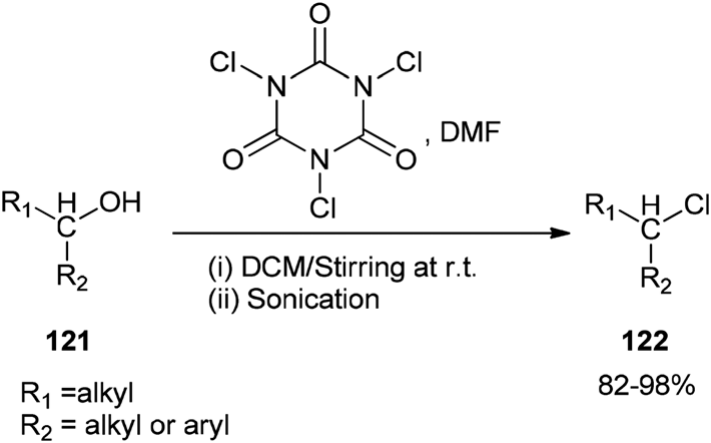
The smooth conversion of alcohols 121 to their corresponding alkyl chlorides 122.

As a mild method, the smooth conversion of alcohols 121 to their corresponding alkyl chlorides 122 were accomplished and reported by Venkanna and co-workers in 2015 in the presence of TCCA/DMF in high yields with a relatively short reaction time (Scheme 58).^142^

**Scheme 59:**
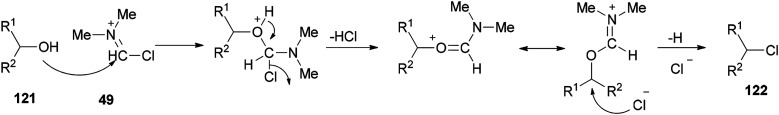
A mechanistic proposal for chlorodehydration using TCCA/DMF.

As shown in Scheme 59, by addition of alcohol 121 to Vilsmeier–Haack (VH) complex 49, an interaction occurs between the hydroxyl group of the alcohol and choloromethyleniminium species (cationic species) which is followed by nucleophilic attack of chloride ion to generate the desired chloride 122. On the basis of observed stereochemical results, the reaction probably occurs through SN_2_ reaction mechanism.

For years, the Vilsmeier reaction has been used to achieve a key transformation such as formylation.^143–146^ There are reports of annulation of aliphatic substances including acyclic ketones,^147–151^ α,β-epoxy ketones,^152^ cyclohexenones,^148,153^ and *etc.*^154–158^ into their aromatic compounds upon treatment with POCl_3_/DMF as the Vilsmeier reagent. Generally, phosgene (COCl_2_) and phosphorus oxychloride (POCl_3_) are toxic reagents which are employed in traditional Vilsmeier–Haack reactions.^159^

**Scheme 60:**
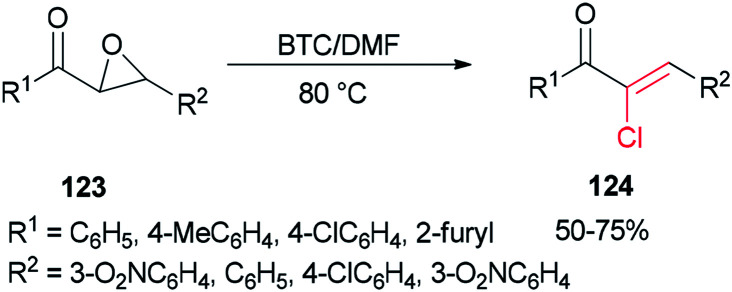
Synthesis of (*Z*)-2-chloro-1,3-diarylpropen-1-ones 124.

Therefore, BTC/DMF (bis(trichloromethyl) carbonate/*N*,*N*-dimethylformamide) adduct was used in 2007 as a Vilsmeier reagent for the effective synthesis of (*Z*)-2-chloro-1,3-diarylpropen-1-ones 124 from 2,3-epoxy-l,3-diarylpropan-1-ones 123, which were readily obtained by oxidation of chalcones using hydrogen peroxide as an oxidant (Scheme 60).^160^ The availability of starting materials and simplicity of manipulation make the present protocol a complement for an attractive synthetic method for academic research and potential applications.

**Scheme 61:**
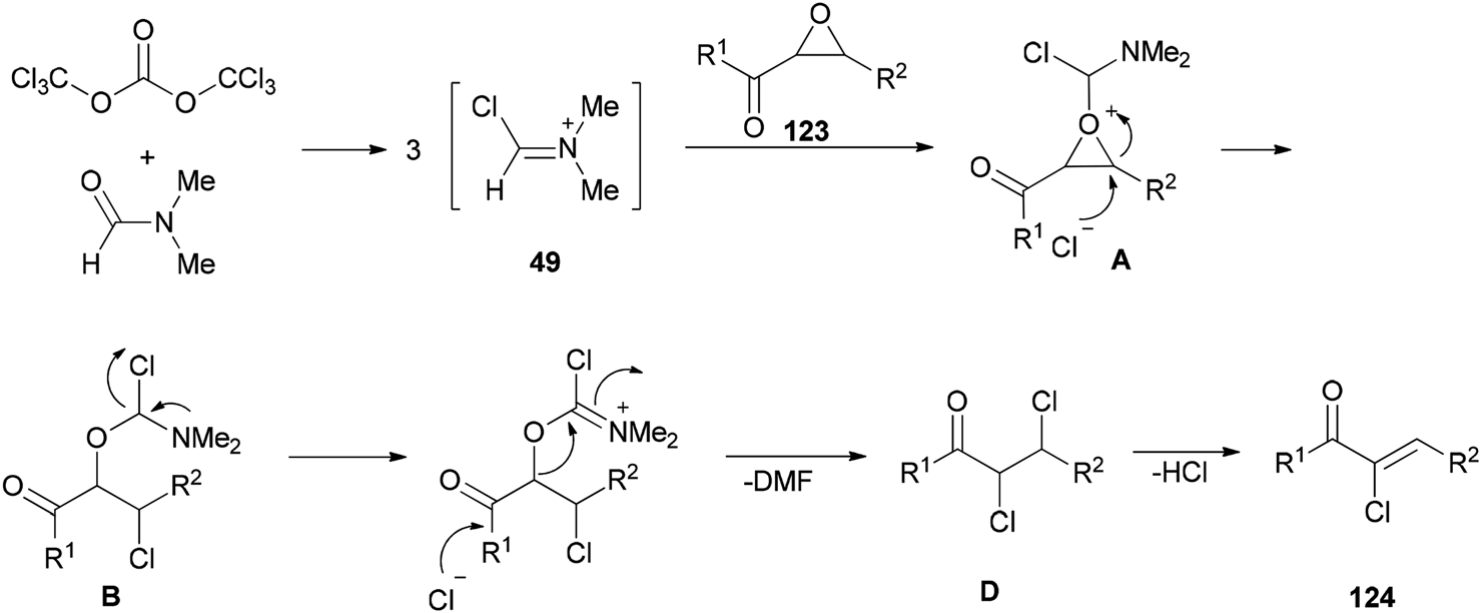
Proposed mechanism for the formation of (*Z*)-2-chloro-1,3-diarylpropen-1-ones 124.

The mechanism of the formation of (*Z*)-2-chloro-1,3-diarylpropen-1-ones 124 could be explained by an initial reaction of BTC with DMF to generate halomethylene iminium salts 49 followed by coordination with 2,3-epoxy-l,3-diarylpropan-1-ones 123 to furnish intermediates oxiranium A, as shown in Scheme 61. Next, intermediates B produced by attack of previously released halide anion from the Vilsmeier reagent on the benzyl position of A led to obtain dichloride D. At the end, one molecule of HCl from compound D is eliminated to yield the desired products 124.

**Scheme 62:**
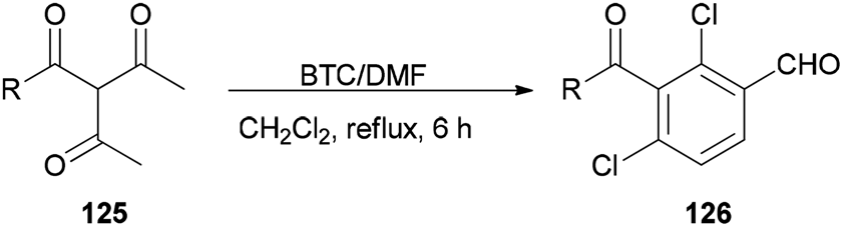
Production of aromatic compounds 126 under Vilsmeier conditions.

Su and co-workers presented a convenient method for the production of a series of substituted aromatic compounds 126 using BTC/DMF as Vilsmeier reagent in refluxing dichloromethane (Scheme 62).^161^ When reaction temperature was increased, decreasing yield and reaction complexity was observed. As an example, the reaction did not occur in DMF. Hence, the best results were obtained by using BTC/DMF in refluxing CH_2_Cl_2_ for 6 h.

**Scheme 63:**
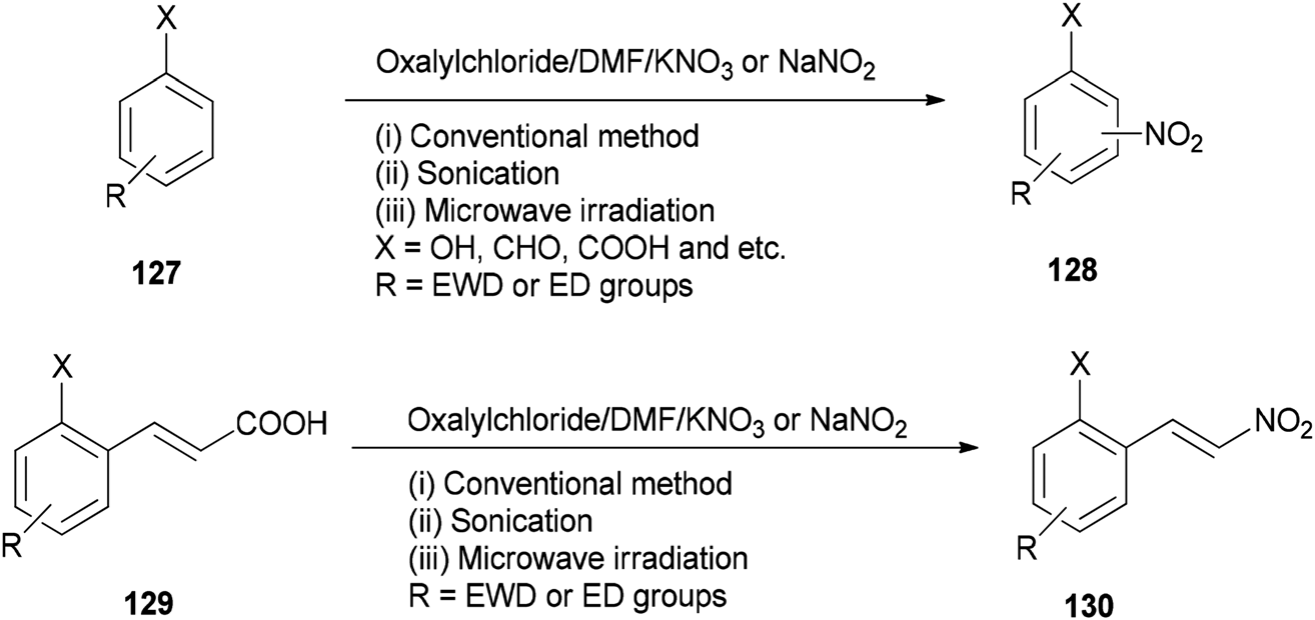
Generation of corresponding nitro derivatives 128 and 130.

As a methodology, generation of the corresponding nitro derivatives 128 and 130 was reported and achieved *via* nitration of aromatic compounds 127 and cinnamic acids 129 with (COCl)_2_/DMF using KNO_3_ or NaNO_2_ under microwave and ultrasonic conditions, respectively (Scheme 63).^162^ In this transformation, the use of microwave and ultrasonic irradiation lead to enhancing the yield of products and reducing reaction time. The main advantages of this present methodology are: (i) work-up procedure is simple; (ii) the products are prepared from readily available and economically cheap reagents; (iii) yields are excellent; and (iv) the reaction time is short.

**Scheme 64:**
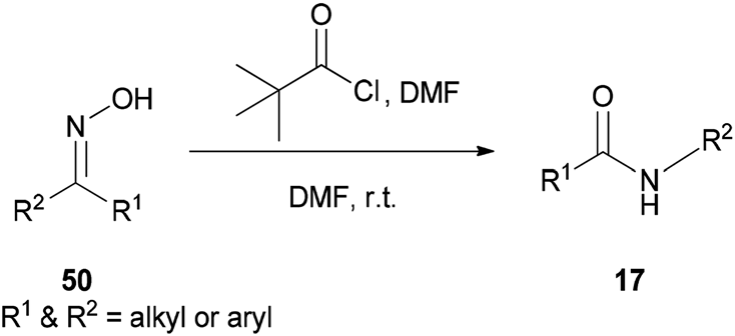
Conversion of ketoximes 50 to their corresponding amides 17.

A new protocol for mild, efficient, and general conversion of ketoximes 50 to their corresponding amides 17 was developed by Narahari and co-workers in 2011 through a Beckmann rearrangement using an inexpensive and nontoxic reagent (pivaloyl chloride/DMF complex) in DMF as the solvent (Scheme 64).^163^ Classical Beckmann rearrangements were generally performed in the presence of strongly acidic and dehydrating media at high temperatures^97,164–166^ while formation of a pivaloyl chloride/DMF complex in this method was achieved at room temperature without the use of acid.

**Scheme 65:**
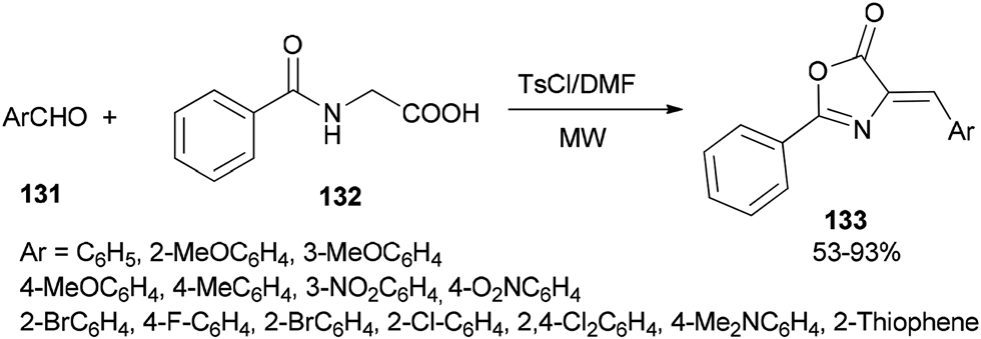
Synthesis of azlactone derivatives 133.

A simple and efficient protocol was designed and reported by Moghanian's group in 2012 for synthesis of azlactone derivatives 133 through a condensation reaction of aromatic aldehydes 131 with hippuric acid 132 using a TsCl and DMF system as condensing agent under microwave irradiation (Scheme 65).^167^ In order to obtain appropriate conditions for this reaction, different bases, such as DMF, pyridine, sodium acetate, and triethylamine were examined in the presence of TsCl under microwave irradiation. Among the bases, DMF gave the best result.

**Scheme 66:**
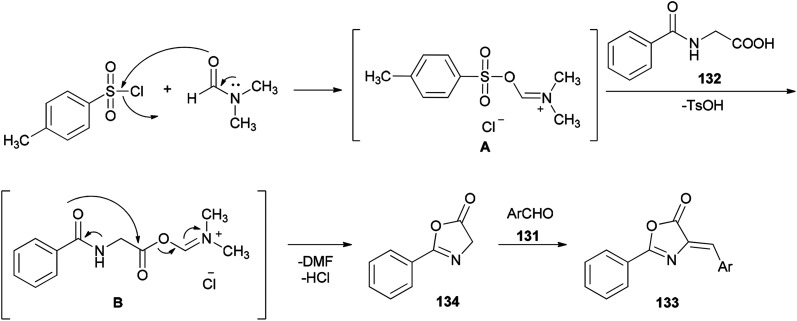
The most possible mechanism for synthesis of azlactone derivatives 133.

Scheme 66 shows a possible mechanism for synthesis of azlactone derivatives 133. The first step involves the formation of the Vilsmeier adduct B from the treatment of hippuric acid 132 with TsCl and DMF. Then, the intermolecular cyclization of intermediate B, produced the azlactone 134 based on good evidence *via* Erlenmeyer synthesis.^168,169^ Finally, aldol condensation of 134 with carbonyl compounds 131 afforded the desired compounds 133.

**Scheme 67:**
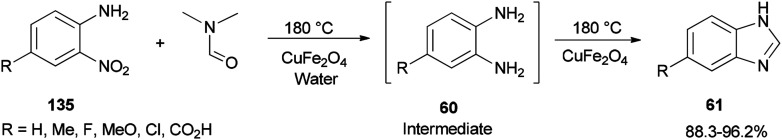
Synthesis of benzimidazoles 61 form *o*-nitroanilines 135.

Quite recently, Yadav and Rasal presented an economical synthetic protocol for preparation of benzimidazoles 61 from *o*-nitroanilines 135 using *N*,*N*-dimethylformamide as the source of C1 and magnetically separable CuFe_2_O_4_ as a cheap and recyclable catalyst in a one pot reaction with 100% conversion in 12 h with 97.5% selectivity (Scheme 67).^170^

**Scheme 68:**
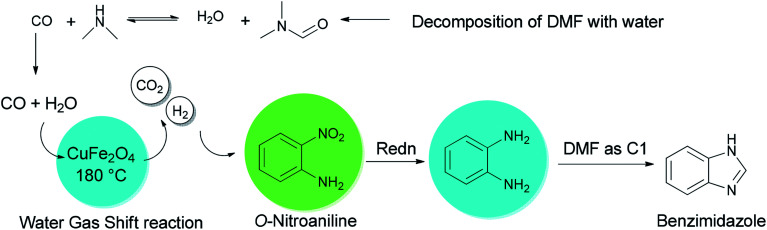
Mechanistic scheme for a one-pot synthesis of benzimidazoles 61.

A mechanistic scheme for a one-pot synthesis of benzimidazoles 61 is shown in Scheme 68. The reaction commences with decomposition of DMF to CO and NMe_2_. Next, H_2_ and CO_2_ are formed from the *in situ* generated CO under water gas shift reaction (WGSR) conditions using CuFe_2_O_4_. The NO_2_ group in *o*-nitroaniline is converted to NH_2_ group in *o*-phenylenediamine *via* reduction using H_2_ and CuFe_2_O_4_. The resulting compound is cyclized in the presence of DMF as a C1 source to afford benzimidazole.

**Scheme 69:**
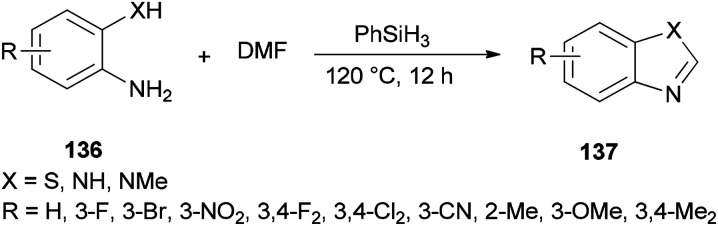
Synthesis of heterocycles 137 from *o*-phenylenediamines 136 and DMF.

Liu and co-workers reported the synthesis of heterocycles 137 from 2-phenylenediamines 136 and DMF using PhSiH_3_ as the only promoter without the need for any other catalysts or additives under metal-free conditions (Scheme 69).^171^

## 3. DMF as a catalyst

Alkoxymethylene-*N*,*N*-dimethyliminium salts were exploited in the catalysis of the Beckmann rearrangement,^172^ preparing 3-acylated indolizines,^173^ and synthesis of benzylidene acetals of mono- and disaccharides.^174^

**Scheme 70:**
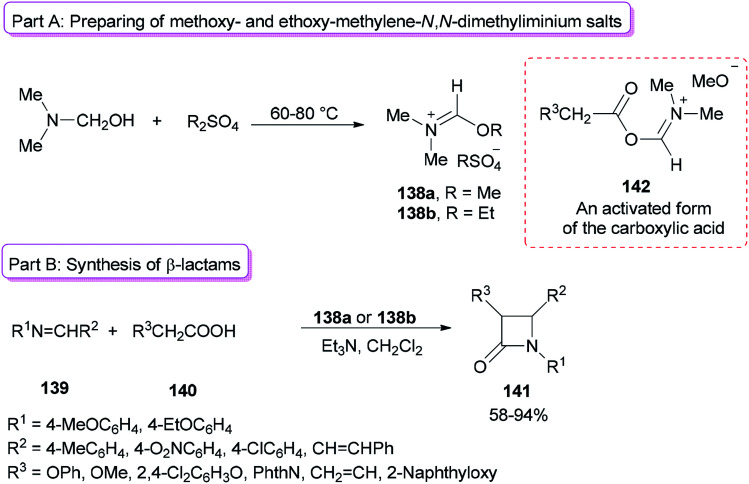
Formation of 2-azetidinones 141.

Formation of 2-azetidinones 141*via* a unique reaction between Schiff bases, substituted acetic acids, and alkoxymethylene-*N*,*N*-dimethyliminium salts was reported by Jarrahpour and co-workers in 2009 (Scheme 70).^175^ The remarkable advantages of this method include low cost, mild reaction conditions, and non-use chlorinating agents as well as easy purification of the products. Treatment of DMF with Me_2_SO_4_ or Et_2_SO_4_ afforded methoxy- or ethoxy-methylene-*N*,*N*-dimethyliminium salts 138a,b (Scheme 70, part A). It should be noted that Me_2_SO_4_ or DMF alone is inactive. The obtained salt 138a from DMF/Me_2_SO_4_ showed better activity than derived salt 138b from DMF/Et_2_SO_4_ (Scheme 70, part B). The best results were gained when DMF and Me_2_SO_4_ were used at room temperature. As a proposal, the reaction just progressed through generation of an activated form of the carboxylic acid 142 which in the following, the corresponding ketene is prepared under deprotonation and loss of DMF from 142.

**Scheme 71:**
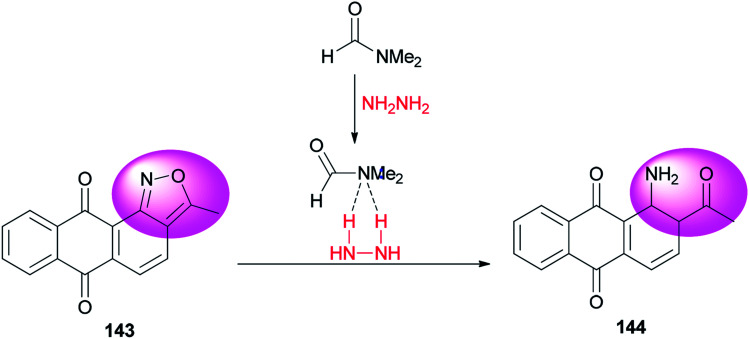
Synthesis of 1-amino-2-acetylanthraquinone 144.

A clean, facile and highly efficient pathway for synthesis of 1-amino-2-acetylanthraquinone 144 (95.0% selectivity) by DMF-promoted reductive ring-cleavage of an isoxazole motif-containing 3-methyl-anthra-[1,2-*c*]-isoxazol-6,11-dion 143 was presented by Zhao and co-workers (Scheme 71).^10^*N*,*N*-Dimethylformamide in this transformation plays dual roles as catalyst and reaction media. The catalytic effect of DMF occurs from formation of a H_2_N–NH_2_/DMF complex by hydrogen bonding.

**Scheme 72:**
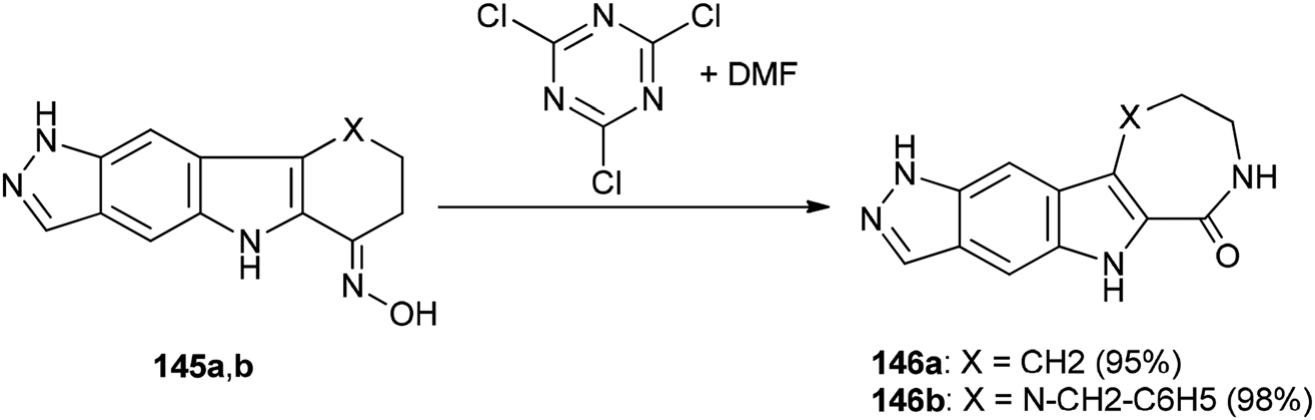
Regioselective synthesis of the corresponding azepine 146a and 1,4-diazepine 146b.

Singh and co-workers in 2013 achieved and reported a very mild protocol for regioselective synthesis of the corresponding azepine 146a and 1,4-diazepine 146b through a Beckmann rearrangement of ketoximes of pyrazolo annulated oxocarbazole 145a and oxoazacarbazole 145b with an organocatalyst derived from DMF and TCTA (Scheme 72).^12^

**Scheme 73:**
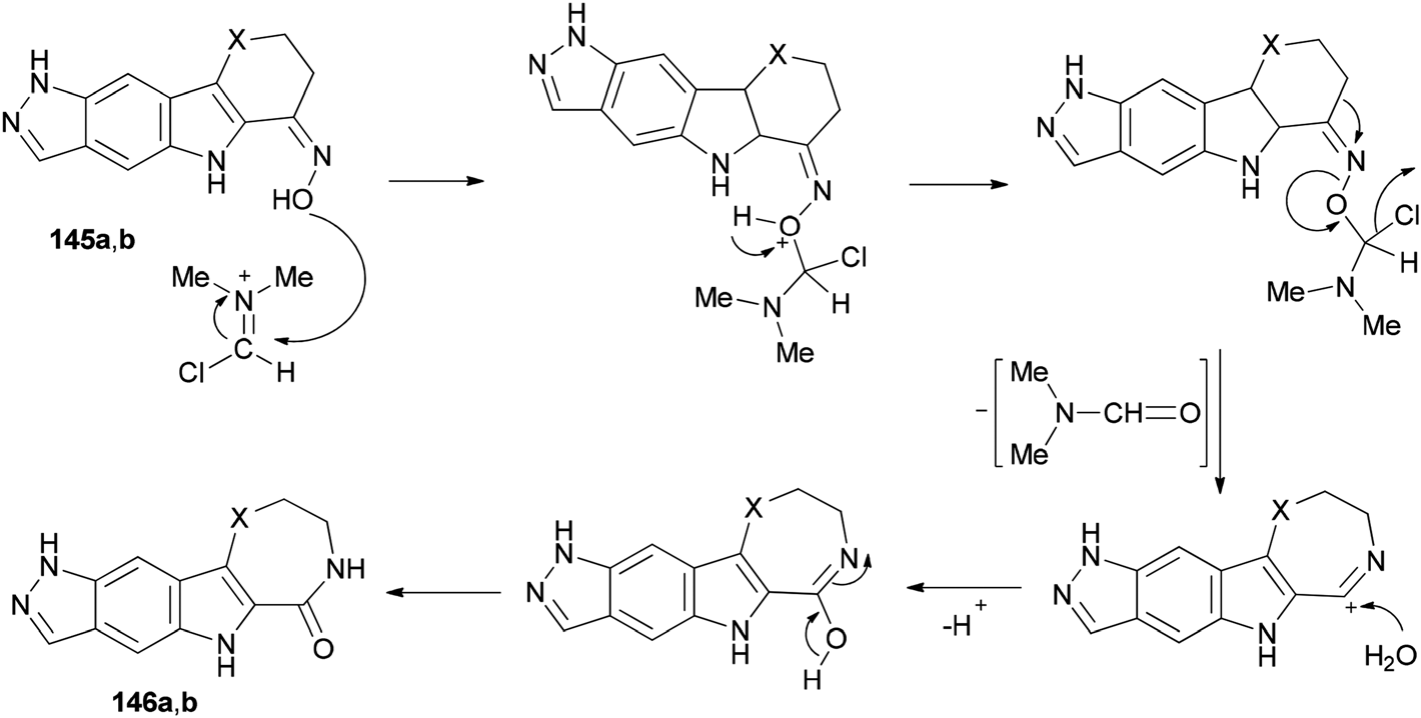
Mechanistic pathway for the formation of 146a,b from 145a,b.

Scheme 73 shows a rational mechanistic pathway for formation of 146a,b from 145a,b. The reaction was carried out through preparation of a complex derived from TCT as a cheap reagent and DMF, that allowed the Beckmann rearrangement to be accomplished with 145a,b to yield 146a,b, respectively.

**Scheme 74:**
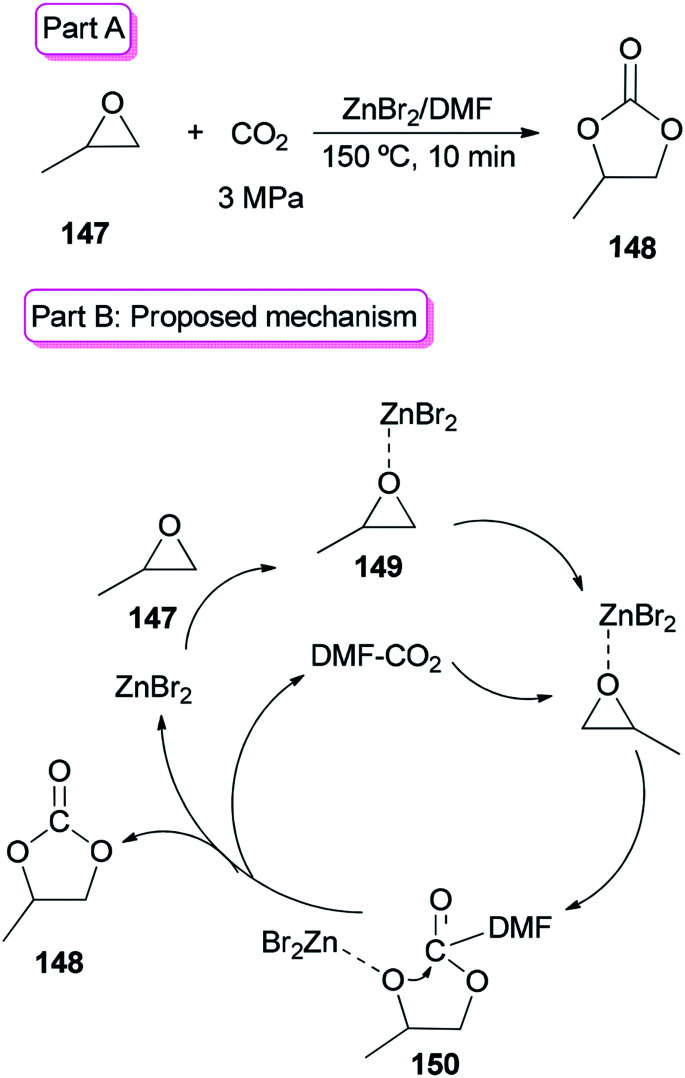
Cycloaddition of propylene oxide 147 with CO_2_ using ZnBr_2_/DMF.

Sun and co-workers prepared the cyclic carbonate 148 by cycloaddition of propylene oxide 147 with CO_2_ catalyzed by ZnBr_2_/DMF in 2005 (Scheme 74, part A).^42^ ZnBr_2_ coupled with a small amount of DMF was converted to highly active species. Interestingly, DMF in a small amount interacted as a co-catalyst with CO_2_ in the gas phase while in a large amount, served as activator and solvent of CO_2_ in the liquid phase. Propylene carbonate 148 was produced in 56.5% yield with 100% selectivity using 3 MPa of CO_2_. The ZnBr_2_/DMF catalyst reported herein compared to the other reported catalysts, is facile available and cost-effective. This catalyst also showed high selectivity/activity, simultaneously. A probable catalytic cycle is proposed in Scheme 74, part B. The reaction began through coordination of PO with ZnBr_2_ to provide the adduct of zinc–epoxide complex 149, which simply gave a propylene oxide open ring; next, the oxy anion of complex 150 was generated by attack of the carboxylate anion (which is formed by CO_2_ activated with DMF) to the less hindered carbon atom of PO; at the late stage, the propylene carbonate 148 is produced by attack of oxy anion to DMF bonded CO_2_ and breaking of the bond Zn–O concurrently, followed by cyclic elimination, in an intermolecular fashion.

**Scheme 75:**
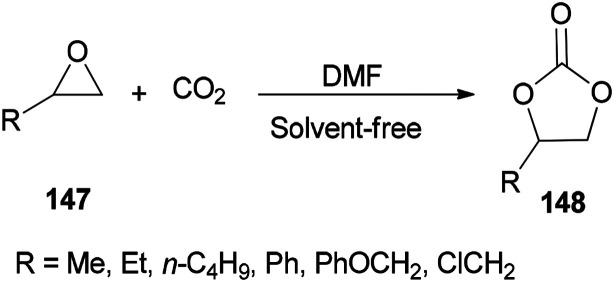
Cycloaddition of epoxides 147 with CO_2_ using DMF under solvent-free conditions.

DMF is a simple, small molecule, and cost-effective organic catalyst which was used for the synthesis of cyclic carbonates 148*via* coupling reaction of epoxides 147 with CO_2_ under solvent-free conditions by Jiang and Hua in 2006 (Scheme 75).^43^

**Scheme 76:**
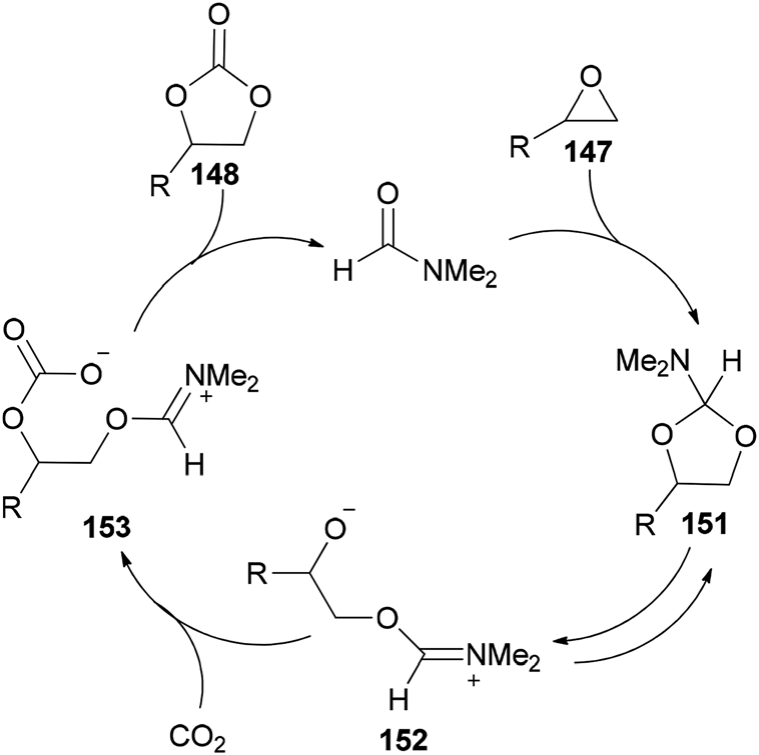
A probable mechanism for cycloaddition of epoxides 147 with CO_2_ using DMF under solvent-free conditions.

Scheme 76 displays a probable mechanism for this transformation. The conversion of 151 to imine salt 152 and then its reaction with CO_2_ afforded the intermediate 153. Finally, the formation of C–O bond provides cyclic carbonate 148 and regenerates DMF.

**Scheme 77:**
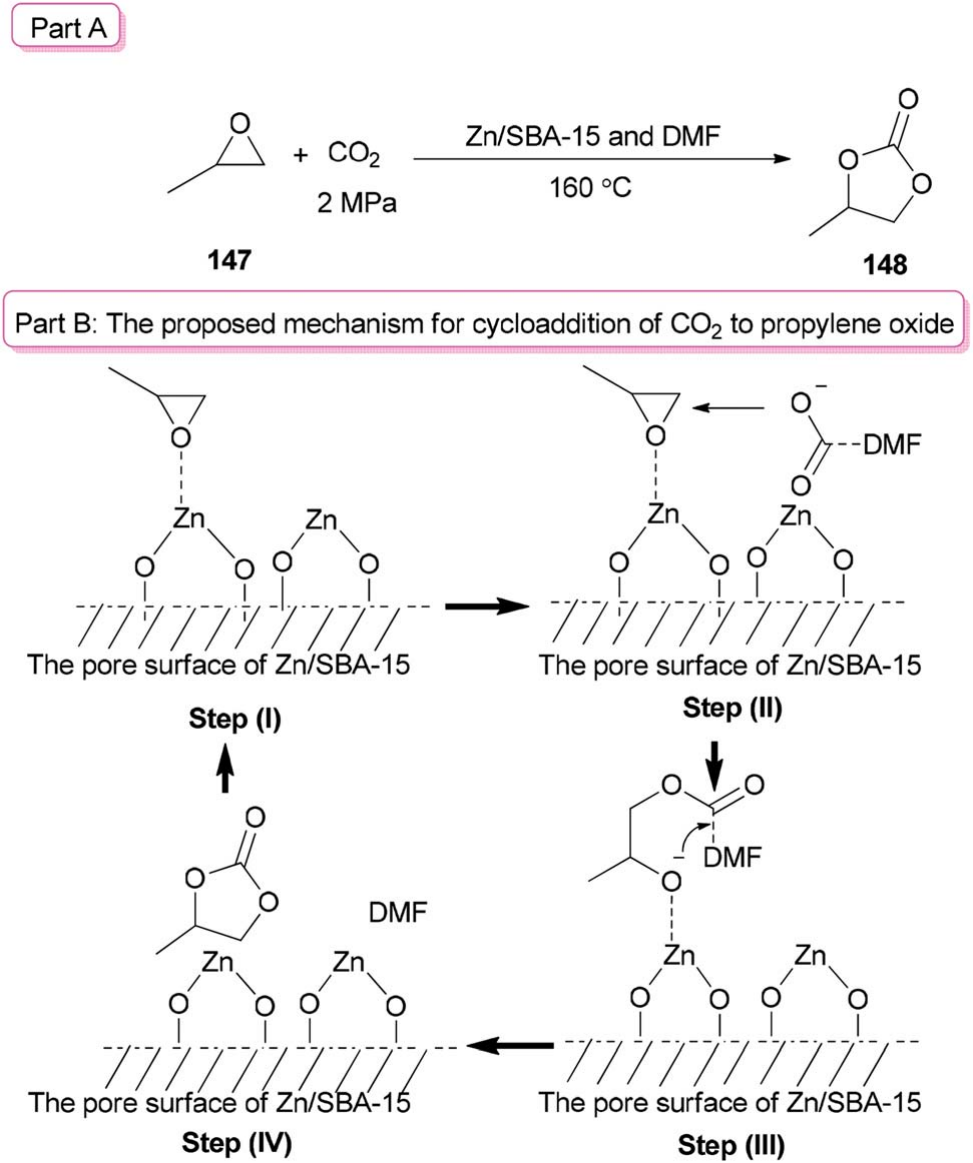
Cycloaddition of epoxides 147 with CO_2_ using Zn/SBA-15 and DMF.

In recent years, metal oxides as inexpensive and simple heterogeneous catalysts have been developed,^176^ while their big draw backs were noticeable.^176–180^ For instance: (1) the use of much higher catalyst/substrate ratios to metal oxides was frequently required; (2) usage of large amounts of a toxic solvent was frequently needed for metal oxides; and (3) they displayed a low activity/selectivity for cycloaddition of CO_2_. To solve problems of the metal oxides, they were loaded onto mesoporous SBA-15. In this method, the surface of metal oxides enhanced the reaction and the amount of metal oxides used decreased. In addition, a small amount of DMF was applied as a catalyst instead of a large amount of organic solvent. Zn/SBA-15 (0.15) as the catalyst formed *via* a one-pot synthetic method was used for cycloaddition reaction of CO_2_ to propylene oxide (PO) 147 in the presence of DMF to give propylene carbonate (PC) 148 in excellent yield (92.3%) (Scheme 77, part A).^41^ A probable mechanism for this reaction is proposed (Scheme 77, part B). In the first step, the Lewis acid center of zinc on the SBA-15 surface activated the propylene oxide. In the second step, the carboxylate anion, which was produced by coordination of nitrogen of DMF with CO_2_, attacked the less sterically hindered carbon atom of PO. As a result, the negative charge of the carboxylate anion was converted to the oxygen atom of PO. The propylene carbonate was generated by intramolecular nucleophilic attack of the oxygen anion on the carbon atom of CO_2_ bonding with DMF (step III) followed by ring-closure reaction (step IV).

**Scheme 78:**
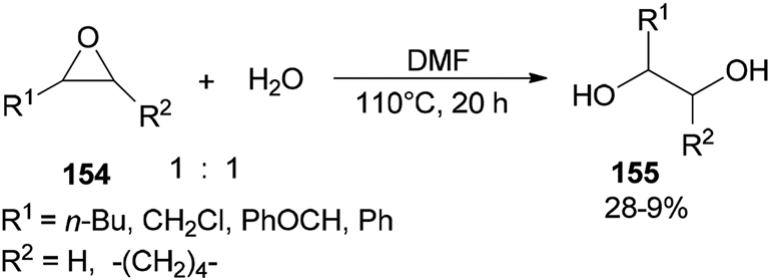
Production of 1,2-diols 155 from epoxides 154 using DMF.

Production of 1,2-diols 155 was performed *via* hydrolysis of epoxides 154 with H_2_O (one equimolar) using DMF. This organocatalytic procedure offers remarkable advantages, such as low cost of the catalyst, mild reaction conditions, and easy separation of the product (Scheme 78).^181^

**Scheme 79:**
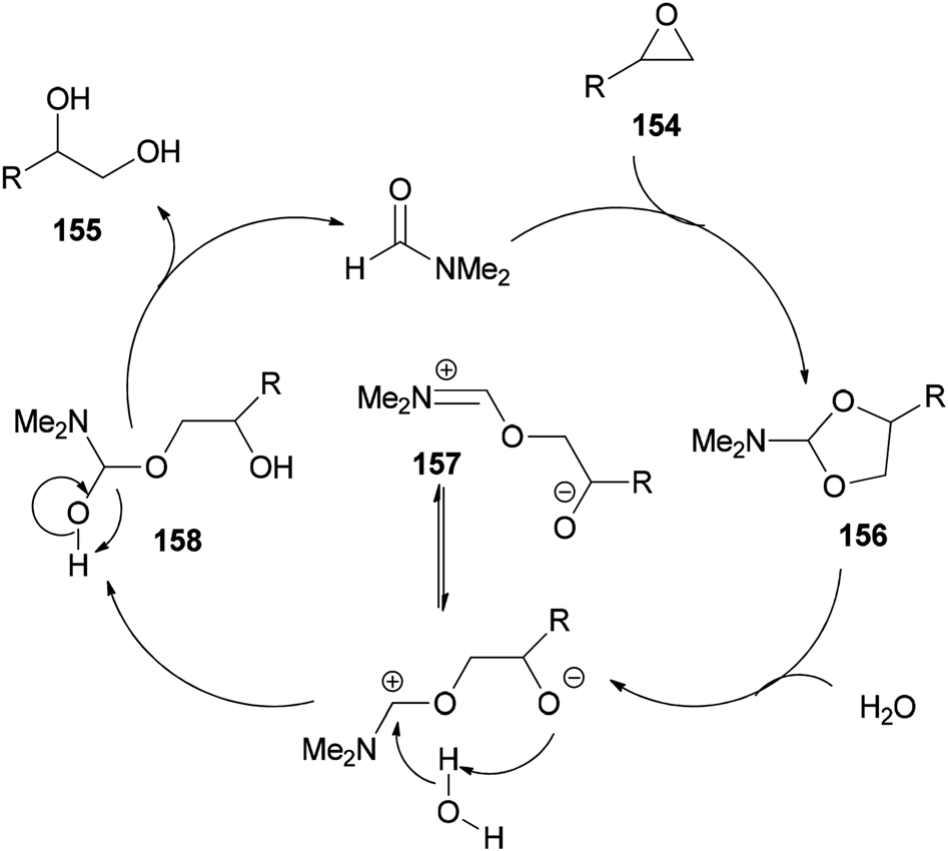
Proposed mechanism for production of 1,2-diols 155 from epoxides 154 using DMF.

As shown in Scheme 79, epoxide 154 is treated with DMF to furnish *N*,*N*-dimethylformamide ethylene acetal derivative 156. The ring of 156 is opened in the presence of H_2_O to give the species 157. The vicinal diol 155 and regenerated DMF are obtained by C–O bond cleavage of intermediate 158 which is, in turn, produced from 157.

**Scheme 80:**
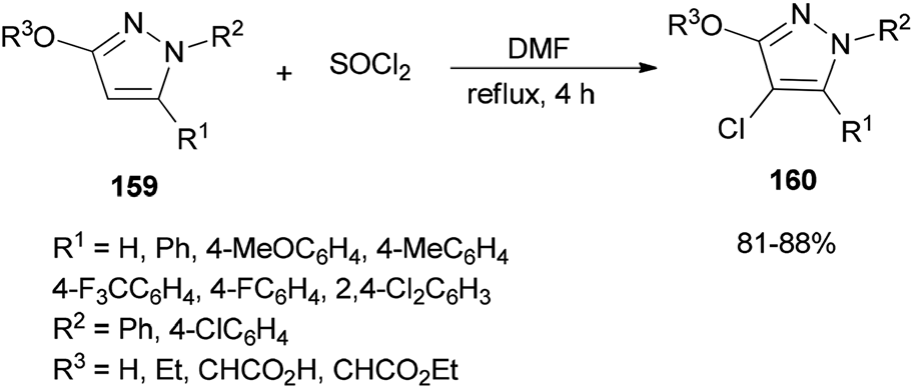
Production of 4-chloro-3-oxypyrazoles 160 from 3-oxypyrazoles 159.

A simple, brief, and efficient method was developed by Zhu and co-workers in 2011 to produce 4-chloro-3-oxypyrazoles 160 by treatment of 3-oxypyrazoles 159 with SOCl_2_ using catalytic amounts of DMF (Scheme 80).^36^ The salient features for this monohalogenation method included high regioselectivity, good functionality tolerance, quick access to products, and experimental simplicity. In this reaction, the catalyst DMF plays a vital role both as an electron-withdrawing group and as a leaving group upon protonation.

**Scheme 81:**
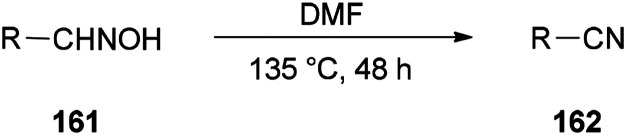
Synthesis of nitriles 162 from aldoximes 161.

Nitriles 162 were obtained by Varvounis and co-workers in 2007 through dehydration of aldoximes 161 by employing DMF as catalyst and solvent (Scheme 81).^182^

**Scheme 82:**
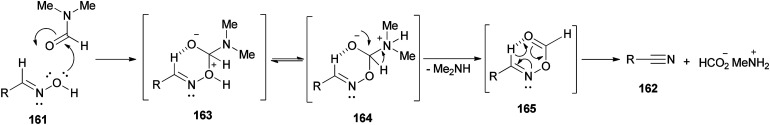
Proposed mechanism for the synthesis of nitriles 162 from aldoximes 161.

It is mechanistically suggested that the reaction occurs by initial attack of the hydroxyl group of the oxime 161 on the carbonyl group of dimethylformamide to yield the cyclic intermediate 163 (Scheme 82). It can lose the proton of hydroxyl to convert to the nitrogen atom of intermediate species 164. Next, the intramolecularly hydrogen bonded aldoxime formate as an intermediate 165 is formed by removal of dimethylamine from 164 which was transformed to the expected nitrile 162 by subsequent thermal elimination of formic acid.

**Scheme 83:**
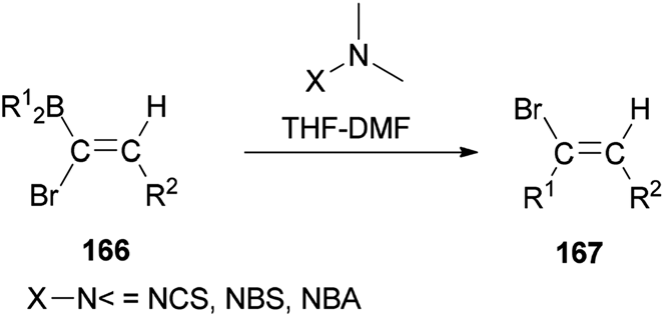
Synthesis of 1,2-disubstituted (*E*)-vinyl bromides 167.

In 2000, and for the first time, a novel and general pathway to synthesize 1,2-disubstituted (*E*)-vinyl bromides 167 was performed by DMF-induced reaction of [(*Z*)-1-bromo-1-alkenyl]dialkylboranes 166 with a *N*-halogeno compound (Scheme 83).^183^ But, it is important to note that this reaction in the absence of *N*,*N*-dimethylmethanamide provided only a trace amount of desired product.

**Scheme 84:**
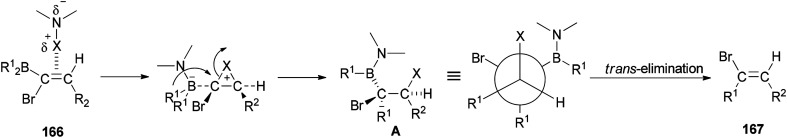
A mechanistic proposal for the synthesis of 167.

A mechanistic proposal for the synthesis of 167 is displayed in Scheme 84. It starts through electrophilic attack of the *N*-halogeno compound. It is supposed that the construction of a halonium ion across the C=C bond is followed by 1,2-migration of an alkyl group from the dialkylboryl group to the α-carbon atom to give intermediate A. Next, the latter under *trans*-elimination produces the 2-disubstituted (*E*)-vinyl bromides 167.

**Scheme 85:**
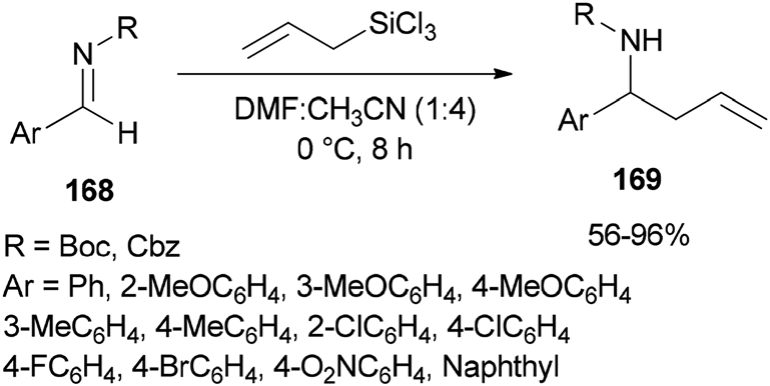
Allylation of various *N*-Boc and *N*-Cbz aldimines 168 with allyltrichlorsilane.

Syntheses of *N*-Boc and *N*-Cbz homoallylic amines 169 was achieved and presented in 2008 by Wu and Sun through facile allylation of various *N*-Boc and *N*-Cbz imines 168 with allyltrichorosilanes as an allylating agent using *N*,*N*-dimethylformamide (dual roles, as an activator and solvent) (Scheme 85).^184^ Interestingly, in the absence of DMF, the reaction was not achieved.

**Scheme 86:**
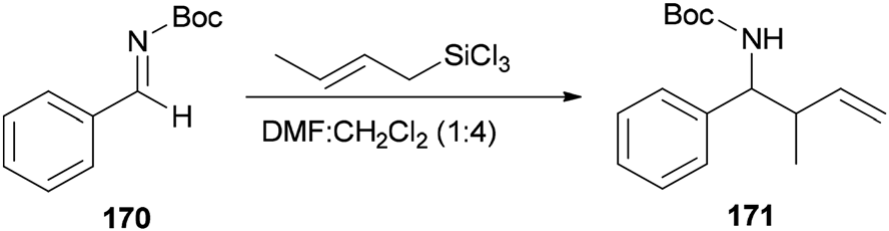
Allylation of *N*-Boc aldimine 170 with (*E*)-crotyltrichlorosilanes.

Wu and Sun also prepared the desired product 171 as a *syn*/*anti* mixture by crotylation of 170 with (*E*)-crotyltrichlorosilanes under conditions of DMF/CH_2_Cl_2_ (1 : 4) (Scheme 86). The performed reaction at −20 °C and 0 °C yielded product 171 in 78% yield (*syn*/*anti* (4 : 6)) and 37% (*syn*/*anti* (5 : 6)), respectively.^184^

**Scheme 87:**
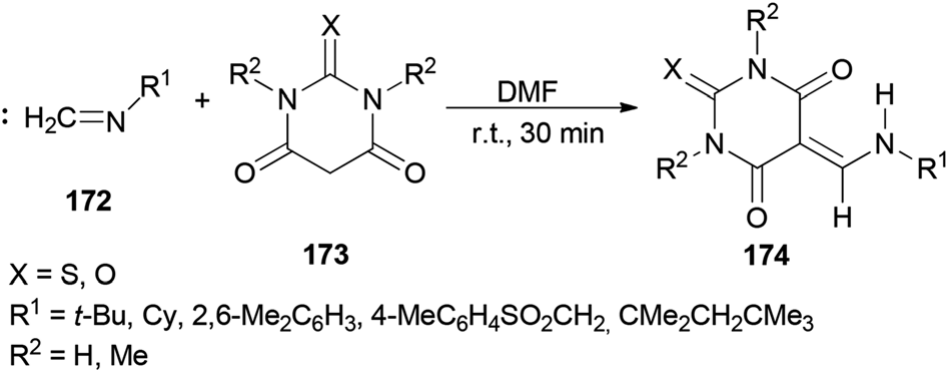
Synthesis of 5-[(alkyl or arylamino)methylene]barbituric acids 174.

In 2010, Teimouri and Tayyebi synthesized 5-[(alkyl or arylamino)methylene]barbituric acids 174 by DMF promoted condensation reactions of alkylisocyanides (or arylisocyanides) 172 with barbituric acid derivatives 173 (Scheme 87).^13^ Compared with the other methods,^185–191^ this methodology has six incomparable advantages: (1) the reaction is simple; (2 and 3) the reaction performs at room temperature and without the use of any other catalyst; (4) yields are good to excellent; (5) products are produced within a short time; and (6) the products are sufficiently pure.

**Scheme 88:**
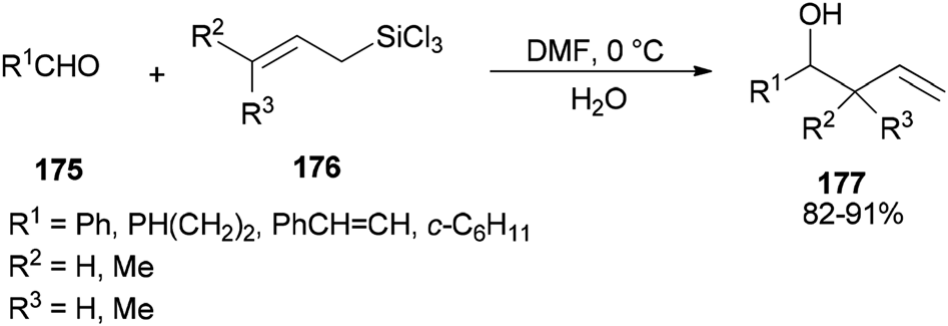
Synthesis of the desired *syn*- and *anti*-homoallylic alcohols 177.

Synthesis of *syn-* and *anti-*homoallylic alcohols 177 was achieved by smooth treatment of aldehydes 175 with (*Z*)- or (*E*)-allyltrichlorosilanes 176 in DMF without any other catalyst, respectively (Scheme 88).^44^

**Scheme 89:**
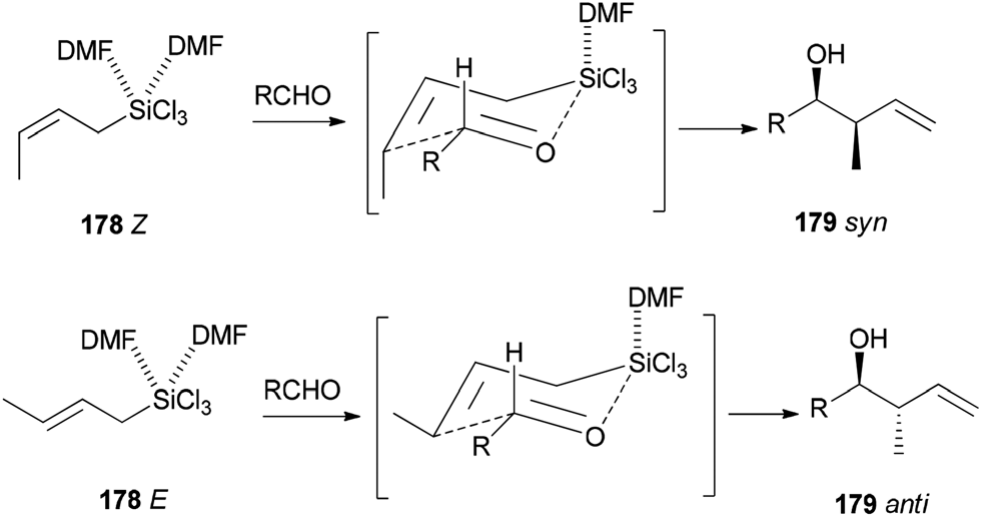
Six-membered cyclic transition state.

Novel C–C bonds were formed only at γ-positions of allyltrlchlorosilanes. As for the transition state, a six-membered molecule is suggested for the allylation of aldehydes (Scheme 89).^192–194^ Among several solvents, DMF is the only solvent that was able to ensue this reaction. The ^29^Si NMR spectra of 1Z showed that *N*,*N*-dimethylformamide coordinated to the Si atom of 1Z to yield the resulting five or six coordinated organosilicate. The organosilicate as a hypervalent molecule has enough Lewis acidity and nucleophilicity, to enable the reaction to proceed smoothly and stereoselectively.

The smooth conversion of β-aminoalcohols to β-amino bromides was accomplished in the presence of DMF and thionyl bromide (SOBr_2_) by Jung and co-workers in 2000.^195^ This method is also applicable for the conversion of both primary and secondary alcohols to their corresponding bromides in high yields.

**Scheme 90:**
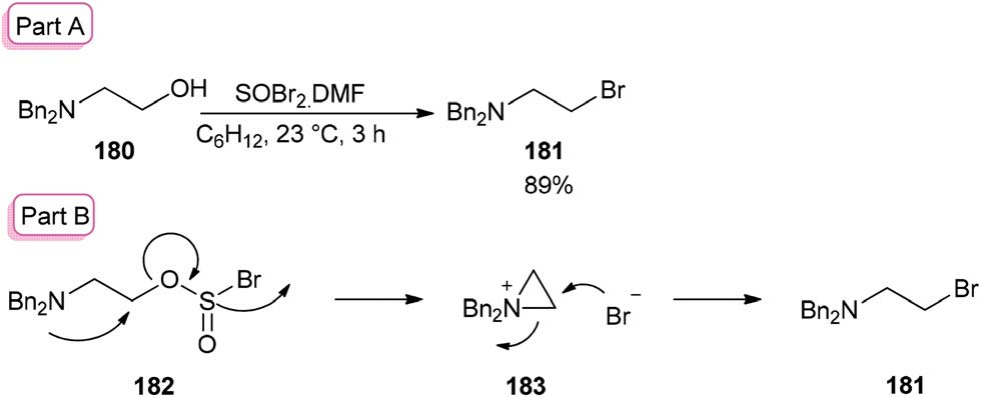
Synthesis of *N*,*N*-dibenzylamino ethanol 181.

As an example, the reaction of *N*,*N*-dibenzylamino ethanol 180 with SOBr_2_ and DMF afforded the expected bromide 181 in excellent (89%) yield and purity (Scheme 90). DMF as a catalyst accelerates the reaction considerably *via* a Vilsmeier–Haack type SOBr_2_–DMF complex. In order to gain appropriate conditions for this method, Jung's group used different polar and nonpolar solvents, such as cyclohexane, hexanes, petroleum ether, dichloromethane, *etc.* Among the solvents, cyclohexane was shown to give the best result.

**Scheme 91:**
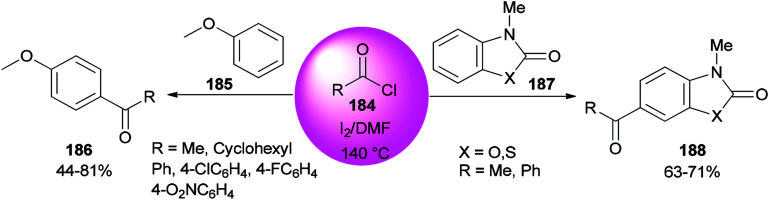
Acylation of anisole 185 and 3-methyl-2(3*H*)-benzoxazolone and 3-methyl-2(3*H*)benzothiazolone substrates 187.

An AlCl_3_–DMF complex was employed as a catalyst in a Friedel–Crafts reaction.^196–198^ In 2008, Poupaert and co-workers presented Friedel–Crafts acylation of aromatic compounds, such as anisole 185, 3-methyl-2(3*H*)-benzoxazolone, and 3-methyl-2(*3H*)-benzothiazolone 187 for synthesis of their aryl ketones using I_2_/DMF as a catalyst. The reaction is carried out under conditions including minimal energy-consumption and minimal wastes. At the end of the reaction, catalyst and wastes are innocuous for the environment (Scheme 91).^46^ The interaction of iodine with the oxygen atom of DMF formed I_2_/DMF as a stable complex. This catalyst exhibits a unique behavior in the Friedel–Crafts acylation of aromatic compounds bearing electron-donating groups.

**Scheme 92:**
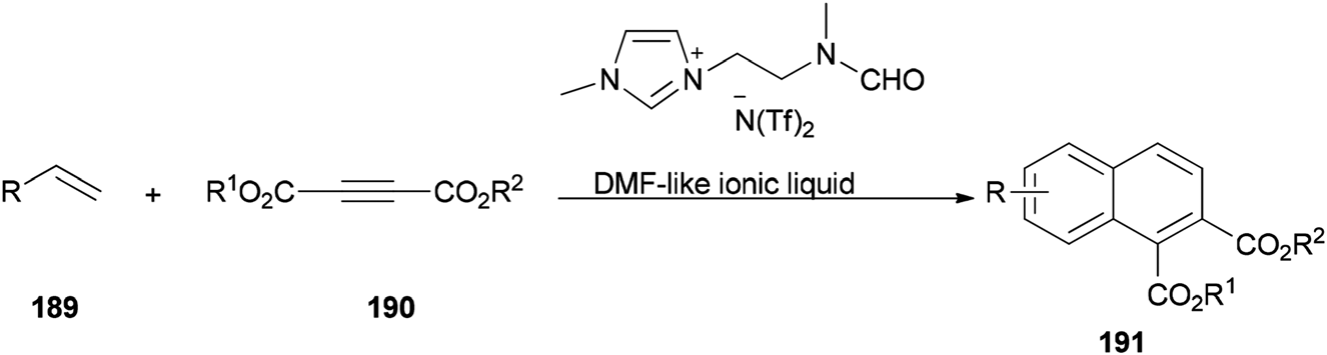
Cycloaddition of vinylarenes 189.

The DMF-based ionic liquid was applied as a nucleophilic reagent and as a solvent for preparing a variety of 1,2 disubstituted-3,4-dihydronaphthalenes 191 by cycloaddition of various vinylarenes 189 with electron-deficient alkynes 190 by Hullio and his group, recently (Scheme 92).^199^

**Scheme 93:**
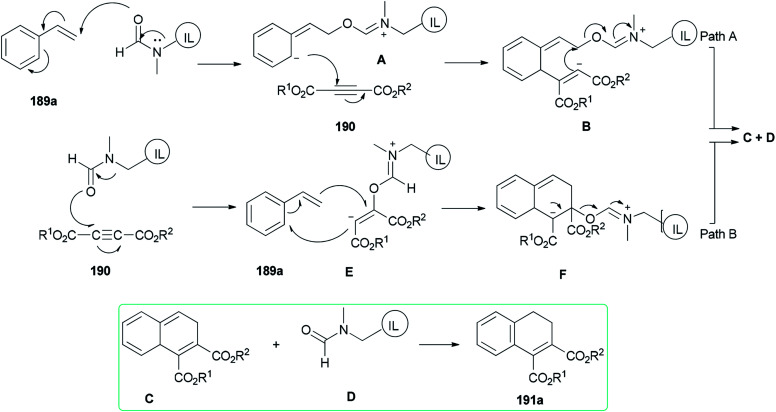
Possible pathways for the cycloaddition of vinylarenes 189a.

Two possible mechanisms are depicted in Scheme 93: (a) nucleophilic attack of the oxygen of DMF-based ionic liquid to the β-carbon of vinylic carbon of styrene 189a to afford the zwitter ionic intermediate A. Next, another zwitter ion intermediate B, which is constructed by nucleophilic attack of the anionic carbon to the acetylenic carbon of acetylene dicarboxylate 190, converted to product C and regenerated ionic liquid D under the indicated bonding arrangements. The corresponding compound A undergoes aromatization to afford the expected product 191a (Scheme 93, Path A). (b) Nucleophilic attack of the oxygen of DMF-based ionic liquid D to the acetylenic carbon of acetylene dicarboxylate 190 to form a highly charged reactive intermediate E. The anionic carbon of E attacks the beta carbon of 189a with the production of another intermediate F that undergoes bond shuffling to obtain C with regeneration of a DMF-based ionic liquid. Finally, the expected product 191a is provided by restoration of aromaticity (Scheme 93, Path B).

## 4. DMF as a stabilizer

**Scheme 94:**
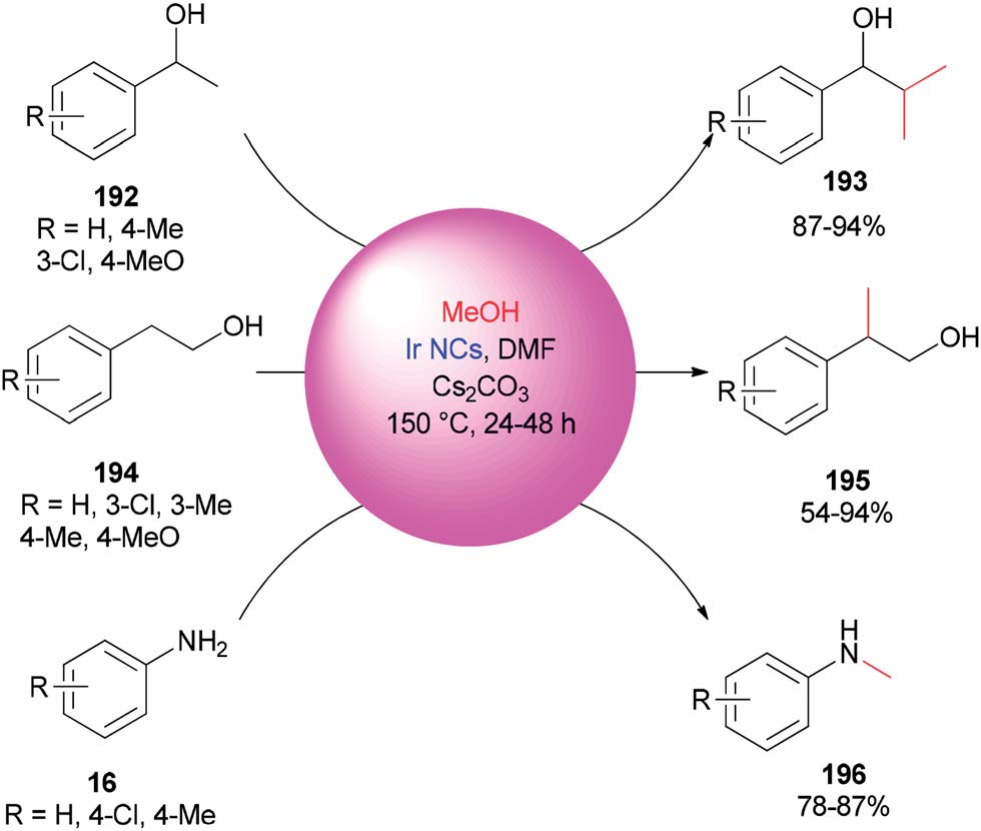
β-Methylation of alcohols 192 and 194 and *N*-methylation of anilines 16 with MeOH.

An alternative and efficient methodology for a solution preparation of DMF-stabilized metal nanoclusters (NCs < 2 nm) and nanoparticles (NPs > 2 nm) of Pt, Au, Cu, and Pd as surfactant-free stable M NCs has been reported.^47,48,50,51^ In this methodology, metal nanoparticles are synthesized using DMF as a solvent, reductant, and stabilizer. The protocol has no need for any external additives. Quite recently, DMF-stabilized Ir nanoclusters were applied as catalysts for methylation of alcohols 192 and 194 and anilines 16 using MeOH as the C1 source by Obora and co-workers (Scheme 94).^53^ These catalysts, which were synthesized in particle sizes of 1–1.5 nm in one step, exhibited an effective catalytic activity in the β-methylation of various primary and secondary alcohols in the presence of MeOH as the source of C1.

**Fig. 2:**
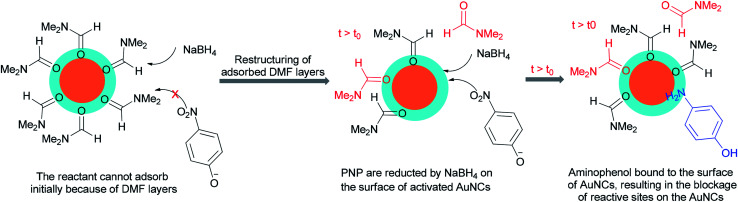
Proposed mechanism for AuNC–catalyzed reduction of 4-nitrophenol to 4-aminophenol.

Kawasaki and co-workers in 2012 investigated the catalytic effect of DMF-stabilized AuNCs in the reduction of 4-nitrophenol (PNP) to 4-aminophenol by sodium borohydride.^11^ A mechanism for AuNC-catalyzed reduction of 4-nitrophenol is suggested as shown in Fig. 2.^11^ Kawasaki proposed that the beginning of the reaction included two steps: initially, the layers of DMF were regenerated, and molecules of DMF partly desorbed from the AuNCs surface. In the second step, PNP penetrated onto the active surface of Au through diffusion. These processes afforded an induction time (*t*_0_), after which the nanoclusters as catalysts facilitated transfer of electrons from BH_4_^−^ to 4-nitrophenol.

**Scheme 95:**
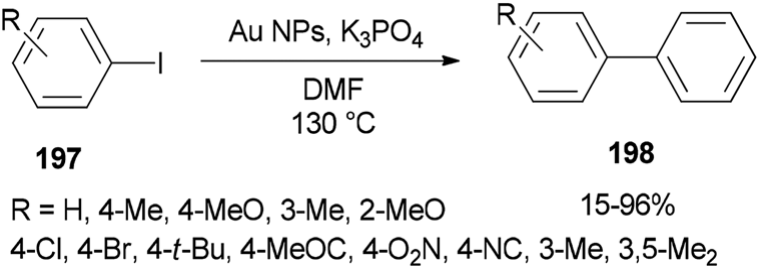
Ullmann homocoupling of aryl iodides 197 catalyzed by DMF-protected Au NPs.

In 2014, Lang and co-workers prepared DMF-stabilized gold nanoparticles by a DMF reduction protocol. By changing reaction time and temperature, the size of the gold nanoparticles could be controlled. Among different sizes of Au NPs, 2.5 nm indicated the highest catalytic activity for Ullmann homocoupling reaction of aryl iodides 197. Apart from DMF, this catalytic coupling reaction does not require any other organic ligand; DMF serves as a reductant, stabilizer, and solvent in the synthesis of metal NP (Scheme 95).^54^

**Scheme 96:**
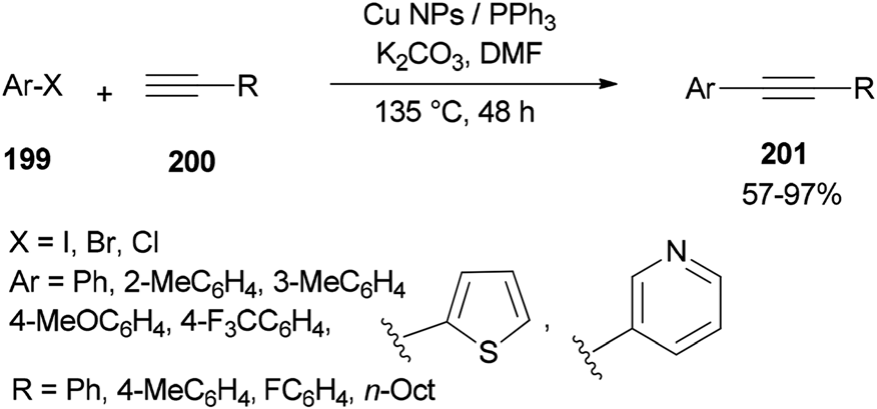
Cu NP-catalyzed Sonogashira cross-coupling reactions of various compounds.

The DMF-stabilized Cu NPs catalyst which was produced exhibited a high catalytic activity in a Sonogashira cross-coupling reaction (Scheme 96).^52^ This synthetic catalyst can also be used in reactions of a wide range of substrates, such as aryl halides 199 and alkynes 200 bearing electron releasing groups (ERG) and electron withdrawing groups (EWG), as well as heterocyclic and sterically hindered substrates. Obora's group found that the reactions could be successfully performed with low loadings of the catalyst.

The Migitae–Kosugie–Stille cross-coupling reaction is based on organostannanes catalyzed by Pd complexes.^200–204^ This reaction was applied in preparing biaryl compounds which are key and vital intermediates in the syntheses of functionalized polymers, pharmaceuticals,^205–208^ and natural products.^209,210^

**Scheme 97:**
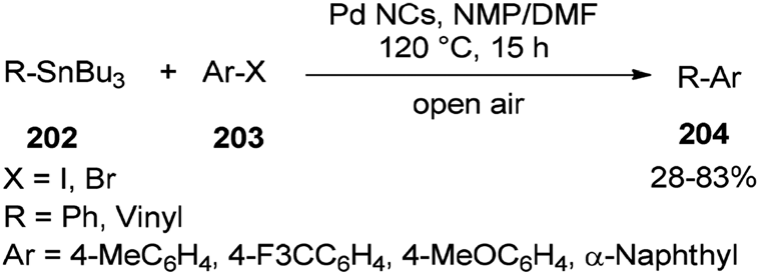
Synthesis of biaryls and vinylarenes 204.

In 2013, synthesis of biaryls and vinylarenes was reported through the Migitae–Kosugie–Stille cross-coupling reaction using *N*,*N*-dimethylformamide-stabilized palladium nanoclusters.^55^ The Migitae–Kosugie–Stille reaction between arylstannane (vinylstannane) 202 with aryl halide 203 in the presence of DMF-stabilized Pd NCs as an efficient and highly active catalyst in the mixture of NMP/DMF (3 : 1) afforded biaryl (vinylarene) 204 (Scheme 97). It was reported that the palladium-catalyzed Migita–Kosugi–Stille reaction is promoted by addition of copper iodide as an additive. Product 204 was provided in excellent yield by addition of CuI.

**Scheme 98:**
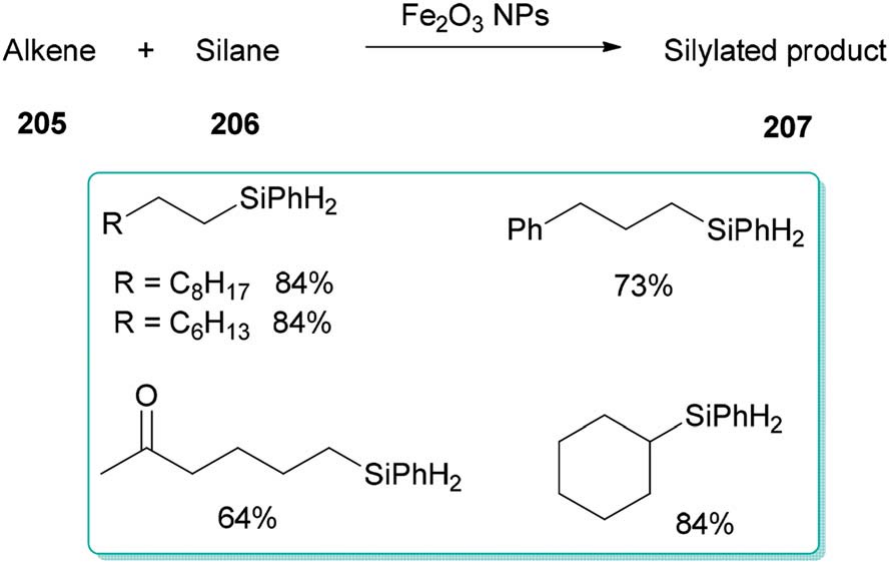
Hydrosilylation of alkenes 205 with hydrosilane 206.

A DMF reduction method was used for production of a monodispersed DMF-stabilized Fe_2_O_3_ NPs (2–5 nm) catalyst by employing Fe(acac)_3_ as a precursor under open air conditions. This novel catalyst displayed efficient catalytic activity for hydrosilylation of an alkene 205 with hydrosilane 206, resulting in versatile silylation products 207, without the need for any additives (Scheme 98).^57^

## 5. Conclusion

This review underscores recent progress in exploiting DMF as a privileged chemical, thanks to its structure. It is a multipurpose compound besides being an effective polar solvent. It acts as a reagent in various reactions such as formylation, amination, aminocarbonylation, amidation, and nitrilation, as well as reactions with arynes. Particularly, employment of DMF as a precursor in nitrilation and formylation reactions have seen important developments. It is noteworthy that in some reactions other amides can be used as precursors as well. With the concern in green and sustainable chemistry, it can be anticipated that application of DMF as a precursor will continue to develop in organic synthesis. Moreover, the development of new catalytic systems with increased reactivity will have important consequences for the practical utilization of DMF as a catalyst. Mechanistic studies in both reported reactions and future developments of novel reactions were conducted. Moreover, applications of DMF as a stabilizer were also discussed. In summary, in this review the main achievements on usage of DMF as a reagent, catalyst, and stabilizer have been summarized and discussed. We hope that it is sufficiently impressive and thorough that it will attract the attention of synthetic organic chemists and will initiate further developments in the applications of DMF beyond being just a polar solvent because it can be so much more with a little innovation.

## Conflicts of interest

There are no conflicts to declare.

## Supplementary Material
